# Range and Speed of Rotor Walks on Trees

**DOI:** 10.1007/s10959-019-00904-1

**Published:** 2019-04-20

**Authors:** Wilfried Huss, Ecaterina Sava-Huss

**Affiliations:** 1 ADB Safegate, Graz, Austria; 2 Institute of Discrete Mathematics, Graz University of Technology, Graz, Austria

**Keywords:** Rotor walk, Range, Rate of escape, Galton–Watson tree, Generating function, Law of large numbers, Recurrence, Transience, Contour function, 05C81, 60J80, 60F05, 60J10

## Abstract

We prove a law of large numbers for the range of rotor walks with random initial configuration on regular trees and on Galton–Watson trees. We also show the existence of the *speed* for such rotor walks. More precisely, we show that on the classes of trees under consideration, even in the case when the rotor walk is recurrent, the range grows at linear speed.

## Introduction

**Fig. 1:**
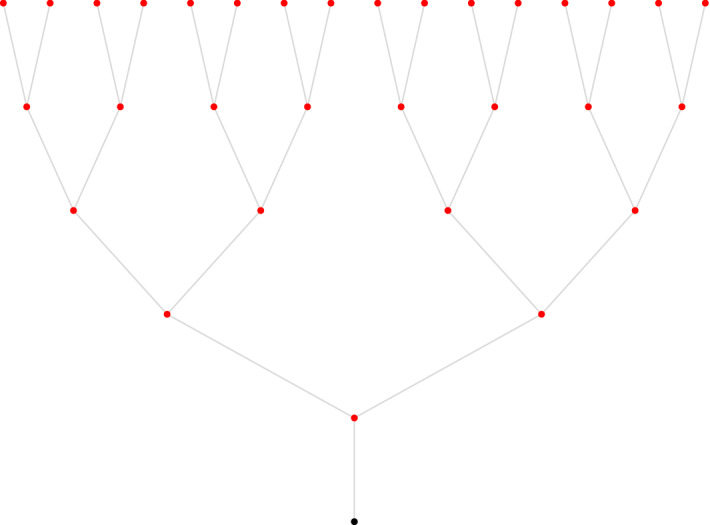
The binary tree ${\mathbb {T}}_2$

For $d\ge 2$, let ${\mathbb {T}}_d$ be the rooted regular tree of degree $d+1$, and denote by *r* the root. We attach an additional * sink vertexo* to the root *r*. We use the notation ${\widetilde{{\mathbb {T}}}}_d = {\mathbb {T}}_d{\setminus }\{o\}$ to denote the tree without the sink vertex. For each vertex $v\in {\widetilde{{\mathbb {T}}}}_d$, we denote its neighbors by $v^{(0)}, v^{(1)},\ldots ,v^{(d)}$, where $v^{(0)}$ is the parent of *v* and the other *d* neighbors, the children of *v*, are ordered counterclockwise (Fig. 1).

Each vertex $v\in {\widetilde{{\mathbb {T}}}}_d$ is endowed with a rotor $\rho (v) \in \{0,\ldots ,d\}$, where $\rho (v)=j$, for $j\in \{0,\ldots ,d\}$ means that the rotor in *v* points to neighbor $v^{(j)}$. Let $(X_n)_{n\in {\mathbb {N}}}$ be a rotor walk on ${\mathbb {T}}_d$ starting in *r* with initial rotor configuration $\rho =(\rho (v))_{v\in {\widetilde{{\mathbb {T}}}}_d}$: for all $v \in {\widetilde{{\mathbb {T}}}}_d$, let $\rho (v)\in \{0,\dots ,d\}$ be independent and identically distributed random variables, with distribution given by ${\mathbb {P}}[\rho (v) = j] = r_j$ with $\sum _{j=0}^dr_j=1$. The rotor walk moves in this way: at time *n*, if the walker is at vertex *v*, then it first rotates the rotor to point to the next neighbor in the counterclockwise order and then it moves to that vertex, that is $X_{n+1}=v^{(\rho (v)+1)\mod (d+1)}$. If the initial rotor configuration is random, then once a vertex has been visited for the first time, the configuration there is fixed. A child $v^{(j)}$ of a vertex $v\in {\mathbb {T}}_d$ is called *good* if $\rho (v)<j$, which means that the rotor walk will first visit the good children before visiting the parent $v^{(0)}$ of *v*. Remark that *v* has $d-\rho (v)$ good children. The *tree of good children* for the rotor walk $(X_n)$, which we denote ${\mathcal {T}}_d^{\mathsf {good}}$, is a subtree of ${\mathbb {T}}_d$, where all the vertices are good children. Let us denote by $R_n = \{X_0, \ldots , X_n\}$ the range on ${\widetilde{{\mathbb {T}}}}_d = {\mathbb {T}}_d{\setminus }\{o\}$ of the rotor walk $(X_n)$ up to time *n*, that is, the set of distinct visited points by the rotor walk $(X_n)$ up to time *n*, excluding the sink vertex *o*. Its cardinality, denoted by $|R_n|$ represents then the number of distinct visited points by the walker up to time *n*.

We denote by $d(r,X_n):=|X_n|$ the distance from the position $X_n$ at time *n* of the rotor walker to the root *r*. The *speed* or the *rate of escape* of the rotor walk $(X_n)$ is the almost sure limit (if it exists) of $\frac{|X_n|}{n}$. We say that $|R_n|$ satisfies a law of large numbers if $\frac{|R_n|}{n}$ converges almost surely to a constant. The aim of this work is to prove a law of large numbers for $|X_n|$ and $|R_n|$, that is, to find constants *l* and $\alpha$ such that

$$\begin{aligned} \lim _{n\rightarrow \infty }\frac{|X_n|}{n}&=l,\quad \text {almost surely},\\ \lim _{n\rightarrow \infty }\frac{|R_n|}{n}&=\alpha ,\quad \text {almost surely}, \end{aligned}$$

when $(X_n)$ is a rotor walk with random initial rotor configuration on a regular tree and on a Galton–Watson tree, respectively. On regular trees, these constants depend on whether the rotor walk $(X_n)$ is recurrent or transient on ${\mathbb {T}}_d$, a property which depends only on the expected value ${\mathbb {E}}[\rho (v)]$ of the rotor configuration at vertex *v*, as shown in [1, Theorem 6]: if ${\mathbb {E}}[\rho (v)]\ge d-1$, then the rotor walk $(X_n)$ is recurrent, and if ${\mathbb {E}}[\rho (v)]< d-1$, then it is transient. Since in the case ${\mathbb {E}}[\rho (v)]=d-1$, the expected return time to the root is infinite, we shall call this case a *critical case*, and we say that the rotor walk is *null recurrent*. Otherwise, if ${\mathbb {E}}[\rho (v)]>d-1$, we say that $(X_n)$ is *positive recurrent*. The tree ${\mathcal {T}}^{\mathsf {good}}_d$ of good children for the rotor walk is a Galton–Watson tree with mean offspring number $d-{\mathbb {E}}[\rho (v)]$ and generating function $f(s)=\sum _{j=0}^{d}r_{d-j}s^j$.

The main results of this paper can be summarized into the two following theorems.

### Theorem 1.1

(Range of the rotor walk) If $(X_n)_{n\in \mathbb {N}}$ is a rotor walk with random initial configuration of rotors on ${\mathbb {T}}_d$, $d\ge 2$, then there exists a constant $\alpha >0$, such that

$$\begin{aligned} \lim _{n\rightarrow \infty }\frac{|R_n|}{n} =\alpha ,\quad \text {almost surely}. \end{aligned}$$

The constant $\alpha$ depends only on *d* and on the distribution of $\rho$ and is given by:

(i) If $(X_n)_{n\in \mathbb {N}}$ is positive recurrent, then
  $$\begin{aligned} \alpha =\frac{d-1}{2{\mathbb {E}}[\rho (v)]}. \end{aligned}$$
(ii) If $(X_n)_{n\in \mathbb {N}}$ is null recurrent, then
  $$\begin{aligned} \alpha =\frac{1}{2}. \end{aligned}$$
(iii) If $(X_n)_{n\in \mathbb {N}}$ is transient, then conditioned on the non-extinction of ${\mathcal {T}}^{\mathsf {good}}_d$,
  $$\begin{aligned} \alpha =\frac{q-f'(q)(q^2-q+1)}{q^2+q-f'(q)(2q^2-q+1)} \end{aligned}$$
  where $q>0$ is the extinction probability of ${\mathcal {T}}^{\mathsf {good}}_d$.

Remark that, even in the recurrent case, the range of the rotor walk grows at linear speed, which is not the case for simple random walks on regular trees. The methods of proving the above result are completely different for the transient and for the recurrent case, and the proofs will be done in separate sections.

### Theorem 1.2

(Speed of the rotor walk) If $(X_n)_{n\in \mathbb {N}}$ is a rotor walk with random initial configuration of rotors on ${\mathbb {T}}_d$, $d\ge 2$, then there exists a constant $l\ge 0$, such that

$$\begin{aligned} \lim _{n\rightarrow \infty }\frac{|X_n|}{n} =l,\quad \text {almost surely}. \end{aligned}$$

(i) If $(X_n)_{n\in \mathbb {N}}$ is recurrent, then $l=0$.
(ii) If $(X_n)_{n\in \mathbb {N}}$ is transient, then conditioned on the non-extinction of ${\mathcal {T}}^{\mathsf {good}}_d$,
  $$\begin{aligned} l=\frac{(q-f'(q))(1-q)}{q+q^2-f'(q)(2q^2-q+1)}, \end{aligned}$$
  where $q>0$ is the extinction probability of ${\mathcal {T}}^{\mathsf {good}}_d$.

The constant *l* is in the following relation with the constant $\alpha$ from Theorem 1.1 in the transient and null recurrent case:

1 $$\begin{aligned} 2\alpha -l=1. \end{aligned}$$

We call this equation the *Einstein relation for rotor walks*. We state similar results for rotor walks on Galton–Watson trees ${\mathcal {T}}$ with random initial configuration of rotors, and we show that in this case, the constants $\alpha$ and *l* depend only on the distribution of the configuration $\rho$ and on the offspring distribution of ${\mathcal {T}}$.

Range of rotor walks and its shape was considered also in [5] on comb lattices and on Eulerian graphs. On combs, it is proven that the size of the range $|R_n|$ is of order $n^{2/3}$, and its asymptotic shape is a diamond. It is conjectured in [7] that on ${\mathbb {Z}}^2$, the range of uniform rotor walks is asymptotically a disk, and its size is of order $n^{2/3}$. In the recent paper [3], for special cases of initial configuration of rotors on transient and vertex-transitive graphs, it is shown that the occupation rate of the rotor walk is close to the Green function of the random walk.

**Organization of the paper.** We start by recalling some basic facts and definitions about rotor walks and Galton–Watson trees in Sect. 2. Then in Sect. 3 we prove Theorem 1.1, while in Sect. 4 we prove Theorem 1.2. Then we prove in Theorem 5.3 and in Theorem 5.5 a law of large numbers for the range and the existence of the speed, respectively, for rotor walks on Galton–Watson trees. Finally, in “Appendix A” we look at the contour function of the range of recurrent rotor walks and its recursive decomposition.

## Preliminaries

### Rotor Walks

Let ${\mathbb {T}}_d$ be the regular infinite rooted tree with degree $d+1$, with root *r*, and an additional vertex *o* which is connected to the root and is called the *sink*, and let ${\widetilde{{\mathbb {T}}}}_d = {\mathbb {T}}_d{\setminus }\{o\}$. Every vertex $v\in {\widetilde{{\mathbb {T}}}}_d$ has *d* children and one parent. For any connected subset $V\subset {\widetilde{{\mathbb {T}}}}_d$, define the set of leaves in ${\widetilde{{\mathbb {T}}}}_d$ as $\partial _o V = \{v \in {\widetilde{{\mathbb {T}}}}_d{\setminus }V: \exists u\in V \text { s. t. } u\sim v\}$ as the set of vertices outside of *V* that are children of vertices of *V*, that is, $\partial _o V$ is the outer boundary of *V*. On ${\widetilde{{\mathbb {T}}}}$, the size of $\partial _o V$ depends only on the size of *V*:

2 $$\begin{aligned} |\partial _o V| = 1+(d-1)| V|. \end{aligned}$$

A *rotor configuration*$\rho$ on ${\widetilde{{\mathbb {T}}}}_d$ is a function $\rho : {\widetilde{{\mathbb {T}}}}_d\rightarrow {\mathbb {N}}_0$, with $\rho (x)\in \{0,\ldots ,d\}$, which can be interpreted as following: each vertex $v\in {\widetilde{{\mathbb {T}}}}_d$ is endowed with a rotor $\rho (v)$ (or an arrow) which points to one of the $d+1$ neighbors. We fix from the beginning a *counterclockwise ordering of the neighbors*$v^{(0)},v^{(1)},\ldots ,v^{(d)}$, which represents the order in which the neighbors of a vertex are visited, where $v^{(0)}$ is the parent of *v*, and $v^{(1)},\ldots ,v^{(d)}$ are the children. A rotor walk $(X_n)$ on ${\mathbb {T}}_d$ is a process where at each time step *n*, a walker located at some vertex $v\in {\widetilde{{\mathbb {T}}}}_d$ first increments the rotor at *v*, i.e., it changes its direction to the next neighbor in the counterclockwise order, and then the walker moves there. We start all our rotor walks at the root *r*, $X_0=r$, with initial rotor configuration $\rho _0=\rho$. Then $X_n$ represents the position of the rotor walk at time *n*, and $\rho _n$ the rotor configuration at time *n*. The rotor walk is also used as *rotor-router walk* in the literature. At each time step, we record not only the position of the walker, but also the configuration of rotors, which changes only at the current position. More precisely, if at time *n* the pair of position and configuration is $(X_n,\rho _n)$, then at time $n+1$ we have

$$\begin{aligned} \rho _{n+1}(x)= {\left\{ \begin{array}{ll} \rho _n(x)+1 \mod (d+1),&{} \text {if } x= X_n\\ \rho _n(x), &{} \text {otherwise}. \end{array}\right. } \end{aligned}$$

and $X_{n+1}=X_n^{(\rho _{n+1}(X_n))}$. As defined above, $(X_n)$ is a deterministic process once $\rho _0$ is determined. Throughout this paper, we are interested in rotor walks $(X_n)$ which start with a *random initial configuration*$\rho _0$ of rotors, which makes $(X_n)$ a random process that is not a Markov chain.

**Random initial configuration.** For the rest of the paper, we consider $\rho$ a random initial configuration on ${\widetilde{{\mathbb {T}}}}_d$, in which $(\rho (v))_{v\in {\widetilde{{\mathbb {T}}}}_d}$ are independent random variables with distribution on $\{0,1,\ldots ,d\}$ given by

3 $$\begin{aligned} {\mathbb {P}}[\rho (v)=j]=r_j, \end{aligned}$$

with $\sum _{j=0}^{d}r_j=1$. If $\rho (v)$ is uniformly distributed on the neighbors, then we call the corresponding rotor walk *uniform rotor walk*. Depending on the distribution of the initial rotor configuration $\rho$, the rotor walk can exhibit one of the following two behaviors: either the walk visits each vertex infinitely often, and it is *recurrent*, or each vertex is visited at most finitely many times, and it escapes to infinity, and this is the *transient case*. For rotor walks on regular trees, the recurrence–transience behavior was proven in [1, Theorem 6], and the proof is based on the extinction/survival of a certain branching process, which will also be used in our results. Similar results on recurrence and transience of rotor walks on Galton–Watson trees have been proven in [6].

For the rotor configuration $\rho$ on ${\widetilde{{\mathbb {T}}}}_d$, a *live path* is an infinite sequence of vertices $v_1,v_2,\ldots$ each being the parent of the next, such that for all *i*, the indices *k* for which $v_{i+1}=v_i^{(k)}$ satisfy $\rho (v)<k$. In other words, $v_1,v_2,\ldots$ is a live path if and only if all $v_1,v_2,\ldots$ are good, and a particle located at $v_i$ will be sent by the rotor walker forward to $v_{i+1}$ before sending it back to the root. An *end* in ${\mathbb {T}}_d$ is an infinite sequence of vertices $o=v_0,v_1,\ldots$, each being the parent of the next. An end is called *live* if the subsequence $(v_i)_{i\ge j}$ starting at one of the vertices $o=v_0,v_1,\ldots$ is a live path. The rotor walk $(X_n)$ can escape to infinity only via a live path.

### Galton–Watson Trees

Consider a Galton–Watson process $(Z_n)_{n\in {\mathbb {N}}_o}$ with offspring distribution $\xi$ given by $p_k={\mathbb {P}}[\xi =k]$. We start with one particle $Z_0 = 1$, which has *k* children with probability $p_k$; then each of these children independently has children with the same offspring distribution $\xi$, and so on. Then $Z_n$ represents the number of particles in the *n*-th generation. If $(\xi _{i}^n)_{i,n\in {\mathbb {N}}}$ are i.i.d. random variables distributed as $\xi$, then

$$\begin{aligned} Z_{n+1}=\sum _{i=1}^{Z_n}\xi _i^n, \end{aligned}$$

and $Z_0=1$. Starting with a single progenitor, this process yields a random family tree ${\mathcal {T}}$, which is called a Galton–Watson tree. The *mean offspring numberm* is defined as the expected number of children of one particle $m={\mathbb {E}}[\xi ]$. In order to avoid trivialities, we will assume $p_0+p_1<1$. The generating function of the process is the function $f(s)=\sum _{k=0}^{\infty }p_ks^k$ and $m=f'(1)$. If is well known that the *extinction probability* of the process, defined as $q=\lim _{n\rightarrow \infty }{\mathbb {P}}[Z_n=0]$, which is the probability the process ever dies out, has the following important property.

#### Theorem 2.1

(Theorem 1, page 7, in [2]) The extinction probability of $(Z_n)$ is the smallest nonnegative root of $s=f(s)$. It is 1 if $m\le 1$ and $<1$ if $m>1$.

With probability 1, we have $Z_n\rightarrow 0$ or $Z_n\rightarrow \infty$ and $\lim _n{\mathbb {P}}[Z_n=0]=1-\lim _n {\mathbb {P}}[Z_n=\infty ]=q$. For more information on Galton–Watson processes, we refer to [2]. When $m<1$, $=1$, or $>1$, we shall refer to the Galton–Watson tree as *subcritical, critical, or supercritical*, respectively.

## Range on Regular Trees

For a simple random walk on a regular tree ${\mathbb {T}}_d$, $d\ge 2$, which is transient, if we denote by $S_n$ its range, then it is known [4, Theorem 1.2] that $|S_n|$ satisfies a law of large numbers:

4 $$\begin{aligned} \lim _{n\rightarrow \infty }\frac{|S_n|}{n} = \frac{d-1}{d}\qquad \text {almost surely}. \end{aligned}$$

We prove a similar result for the range of any rotor walk with random initial configuration on a regular tree ${\mathbb {T}}_d$. From [1, Theorem 6], $(X_n)$ is recurrent if ${\mathbb {E}}[\rho (v)] = \sum _{j=1}^d j r_j \ge d - 1$ and transient if ${\mathbb {E}}[\rho (v)]<d-1$. The tree of good children for the rotor walk, denoted ${\mathcal {T}}^{\mathsf {good}}_d$ and defined in Sect. 2.1, is then a Galton–Watson process with offspring distribution $\xi =$ number of good children of a vertex, given by

$$\begin{aligned} {\mathbb {P}}[\xi =j]=r_{d-j}, \text { for } j=0,1,\ldots d. \end{aligned}$$

Each vertex has, independently of all the others, a number of good children with the same distribution $\xi$. The mean offspring number of ${\mathcal {T}}^{\mathsf {good}}_d$ is $m=d-{\mathbb {E}}[\rho (v)]$. Let $f(s)=\sum _jr_{d-j}s^j$ be the generating function for ${\mathcal {T}}^{\mathsf {good}}_d$.

The lemma below is a key observation that is crucial for the main results of this paper. For a proof, we refer to [1].

### Lemma 3.1

Let ${\mathcal {T}}^{\mathsf {good}}_d$ be the tree of good children of the sink vertex of the current rotor configuration. Then for every excursion (i.e., a rotor walk that is started at the sink vertex and is stopped the first time it returns to the sink vertex), we have the following:

- If the component of the sink vertex in ${\mathcal {T}}^{\mathsf {good}}_d$ is finite, then every vertex *v* in this component will be visited in the excursion exactly $d+1-{\mathbb {E}}[\rho (v)]$ times.
- If the component of the sink vertex in ${\mathcal {T}}^{\mathsf {good}}_d$ is infinite, then the walker will escape through the rightmost live path. Furthermore, every vertex to the right of this live path will be visited exactly $d+1-{\mathbb {E}}[\rho (v)]$ times.

For proving Theorem 1.1, we shall treat the three cases separately: the positive recurrent, null recurrent and transient case.

### Recurrent Rotor Walks

In this section, we consider recurrent rotor walks $(X_n)$ on ${\mathbb {T}}_d$, that is, once again from [1, Theorem 6] ${\mathbb {E}}[\rho (v)] \ge d - 1$. Then ${\mathcal {T}}^{\mathsf {good}}_d$ has mean offspring number $m\le 1$, which by Theorem 2.1 dies out with probability one. While in the case $m<1$, where the rotor walk is positive recurrent, the expected size of ${\mathcal {T}}^{\mathsf {good}}_d$ is finite, this is not the case when $m>1$. For this reason, we handle these two cases separately. In order to prove a law of large numbers for the range $R_n= \{X_0,X_1,\ldots , X_n\}$ of the rotor walk up to time *n*, we first look at the behavior of the rotor walk at the times when it returns to the sink *o*. Define the times $(\tau _k)$ of the *k*-th return to the sink *o*, by: $\tau _0 = 0$ and for $k\ge 1$ let

5 $$\begin{aligned} \tau _k = \inf \{n > \tau _{k-1}: X_n = o\}. \end{aligned}$$

At time $\tau _{k}$, the walker is at sink, all rotors in the visited set $R_{\tau _k}$ point toward the root, while all other rotors still are in their initial configuration. Between the two consecutive stopping times $\tau _{k-1}$ and $\tau _k$, the rotor walk performed a depth first search in the finite subtree induced by $R_{\tau _k}$, by visiting every child of a vertex in right to left order. For every vertex in $v\in R_{\tau _k}$, we can uniquely associate the edge $(v,v^{(0)})$, with $v^{(0)}$ being the unique ancestor of *v*, which implies that $|R_{\tau _k}|$ equals the number of edges in the tree induced by $R_{\tau _k}$. In a depth first search of $R_{\tau _k}$, each edge is visited exactly two times, and in view of the bijection above, it requires exactly $2|R_{\tau _k}|$ steps to return to the origin. We can then deduce that

6 $$\begin{aligned} \tau _{k} - \tau _{k-1} = 2 |R_{\tau _k}|. \end{aligned}$$

Since we are in the recurrent case, where the rotor walk returns to the sink infinitely many times, these stopping times are almost surely finite.

#### Positive Recurrent Rotor Walks

If ${\mathbb {E}}[\rho (v)] > d - 1$, we prove the following.

##### Theorem 3.2

For a positive recurrent rotor walk $(X_n)$ on ${\mathbb {T}}_d$, with $d\ge 2$ we have

$$\begin{aligned} \lim _{k\rightarrow \infty }\frac{| R_{\tau _k}|}{\tau _k} = \frac{d-1}{2{\mathbb {E}}[\rho ]}, \quad {\text {almost surely}}. \end{aligned}$$

##### Proof

For simplicity of notation, we write ${\mathcal {R}}_k = R_{\tau _k}$. The tree of good children, ${\mathcal {T}}^{\mathsf {good}}_d={\mathcal {R}}_1$ is a subcritical Galton–Watson tree with mean offspring number $m = d - {\mathbb {E}}[\rho ]<1$, that is, it dies out almost surely. The expected size of the range up to time $\tau _1$ is given by ${\mathbb {E}}[|{\mathcal {R}}_1|] = \frac{1}{1-m}>1$. At the time $\tau _k$ of the *k*-th return to the sink *o*, all rotors in the previously visited set ${\mathcal {R}}_k$ point toward the root, and the remaining rotors are still in their initial configuration. Thus, during the time interval $(\tau _k, \tau _{k+1}]$, the rotor walk visits all the leaves of ${\mathcal {R}}_k$ from right to left, and at each leaf it attaches independently a (random) subtree that has the same distribution as ${\mathcal {R}}_1$. If we denote by $L_k = |\partial _o {\mathcal {R}}_k|$, then $L_{k+1} = \sum _{i=1}^{L_k} L_{1,i}$, where $L_{1,i}$ are independent copies of $L_1$, and $(L_k)$ is a supercritical Galton–Watson process with mean offspring number $\nu$ (the mean number of leaves of $|{\mathcal {R}}_1|$), which in view of (2), is given by

$$\begin{aligned} \nu = 1+(d-1){\mathbb {E}}[|{\mathcal {R}}_1|]=1 + \frac{d-1}{1-m}>1. \end{aligned}$$

Moreover, ${\mathbb {P}}[L_k = 0] = 0$. Since $\nu >1$, it follows from the Seneta–Heyde Theorem (see [8]) applied to the supercritical Galton–Watson process $(L_k)$ that there exists a sequence of numbers $(c_k)_{k\ge 1}$ and a nonnegative random variable *W* such that

(i) $$\displaystyle \frac{L_k}{c_k} \rightarrow W,\quad \text {almost surely.}$$
(ii) $\displaystyle {\mathbb {P}}[W = 0]$ equals the probability of extinction of $(L_k)$.
(iii) $$\displaystyle \frac{c_{k+1}}{c_k} \rightarrow \nu $$.

By (2) we have $|{\mathcal {R}}_k| = \frac{L_k - 1}{d-1}$ almost surely, which together with Eq. (6) yields

$$\begin{aligned} (d-1)\big (\tau _k - \tau _{k-1}\big ) + 2 = 2(d-1) |{\mathcal {R}}_k| + 2 = 2 L_k,\quad \text {almost surely}, \end{aligned}$$

and dividing by $c_k$ gives

$$\begin{aligned} (d-1)\left( \frac{\tau _k}{c_k} - \frac{\tau _{k-1}}{c_{k-1}}\cdot \frac{c_{k-1}}{c_k}\right) +\frac{2}{c_k} = 2\frac{L_k}{c_k},\quad \text {almost surely}. \end{aligned}$$

It then follows that

$$\begin{aligned} \left( \frac{\tau _k}{c_k} - \frac{\tau _{k-1}}{c_{k-1}}\cdot \frac{c_{k-1}}{c_k}\right) = \frac{2}{d-1} \frac{L_k}{c_k} - \frac{2}{(d-1) c_k}\xrightarrow [k\rightarrow \infty ]{} \frac{2}{d-1} W, \end{aligned}$$

since $c_k\rightarrow \infty$. Since $\frac{\tau _k}{c_k}$ and $\frac{\tau _{k-1}}{c_{k-1}}$ either both diverge or have the same limit $\tau ^\star$, it follows that

$$\begin{aligned} \left( \tau ^\star - \frac{\tau ^\star }{\nu }\right) = \frac{2}{d-1} W. \end{aligned}$$

Thus, $\frac{\tau _k}{c_k}$ converges almost surely to an almost surely positive random variable $\tau ^\star$

$$\begin{aligned} \tau ^\star = \lim _{k\rightarrow \infty }\frac{\tau _k}{c_k} = \left( 1-\frac{1}{\nu }\right) ^{-1} \frac{2}{d-1} W > 0. \end{aligned}$$

Hence,

$$\begin{aligned} \frac{\tau _{k-1}}{\tau _k} = \frac{\tau _{k-1}}{c_{k-1}}\cdot \frac{c_{k-1}}{c_k} \cdot \frac{c_k}{\tau _k} \xrightarrow [k\rightarrow \infty ]{} \tau ^\star \cdot \frac{1}{\nu } \cdot \frac{1}{\tau ^\star } = \frac{1}{\nu },\quad \text { almost surely.} \end{aligned}$$

Now from (6), we get

$$\begin{aligned} \lim _{k\rightarrow \infty } \frac{|{\mathcal {R}}_k|}{\tau _k}&= \lim _{k\rightarrow \infty } \frac{1}{2}\left( 1- \frac{\tau _{k-1}}{\tau _k}\right) =\frac{1}{2}\left( 1-\frac{1}{\nu }\right) = \frac{d-1}{2(d-m)} = \frac{d-1}{2{\mathbb {E}}[\rho (v)]}, \end{aligned}$$

and this proves the claim. $\square$

Passing from the range along a subsequence $(\tau _k)$ to the range $R_n$ at all times requires additional work, because of the exponential growth of the increments $(\tau _{k+1}-\tau _k)$. We next prove that the almost sure limit $\frac{|R_n|}{n}$ exists.

##### Proof of Theorem 1.1(i)

Let $x_1,\ldots ,x_d$ be the *d* children of the root vertex of ${\mathbb {T}}_d$. Let $R_k^{(1)},\ldots ,R_k^{(d)}$ be the range of *d* independent recurrent rotor walks on the tree ${\mathbb {T}}_d$ with i.i.d. initial rotor configurations, at the *k*-th return to the sink vertex. Moreover, let $\xi _k^{(1)},\ldots ,\xi _k^{(d)}$ be the times of the *k*-th visit to the sink vertex by these rotor walks. One can couple the original rotor walk with these *d* independent rotor walks in such a way that the dynamics of the original rotor walk in the component of the tree rooted at $x_i$ is given by the dynamics in the *i*-th rotor walk, for $i=1,\ldots ,d$.

Let *n* be an arbitrary positive number in $(\tau _k,\tau _{k+1})$. Then the original rotor walk $(X_n)$ at time *n* is located in the component of the tree rooted at $x_i$ for some *i*. The coupling above then gives us these two inequalities:

$$\begin{aligned} n&\ge \xi _{k+1}^{(1)}+\cdots +\xi _{k+1}^{(i-1)}+\xi _k^{(i)}+\cdots +\xi _k^{(d)}+k\\ R_n&\le R_{k+1}^{(1)}+\cdots +R_{k+1}^{(i)}+R_k^{(i+1)}+\cdots +R_k^{(d)}+k. \end{aligned}$$

Note that the addition of *k* in the inequalities above is to account for the time spent at the sink vertex in the original rotor walk. The contribution of *k* is negligible as $n\rightarrow \infty$ as far as we are concerned. It then follows that

$$\begin{aligned} \limsup _{n\rightarrow \infty }\frac{R_n}{n}\le \limsup _{n\rightarrow \infty }\dfrac{R_{k+1}^{(1)}+\cdots +R_{k+1}^{(i)}+R_k^{(i+1)}+\cdots +R_k^{(d)}}{\xi _{k+1}^{(1)}+\cdots +\xi _{k+1}^{(i-1)}+\xi _k^{(i)}+\cdots +\xi _k^{(d)}}, \end{aligned}$$

where $k:=k(n)$ and $i:=i(n)$ depend on *n*. By Theorem 3.2, it then follows that

$$\begin{aligned} \limsup _{n\rightarrow \infty }\frac{R_n}{n}\le \limsup _{n\rightarrow \infty }\alpha \dfrac{\xi _{k+1}^{(1)}+\cdots +\xi _{k+1}^{(i)}+\xi _k^{(i+1)}+\cdots +\xi _k^{(d)}}{\xi _{k+1}^{(1)}+\cdots +\xi _{k+1}^{(i-1)}+\xi _k^{(i)}+\cdots +\xi _k^{(d)}}, \end{aligned}$$

for $\alpha =\frac{d-1}{2{\mathbb {E}}[\rho ]}$. A direct calculation then gives us

7 $$\begin{aligned} \limsup _{n\rightarrow \infty }\frac{R_n}{n}\le \alpha +\alpha \limsup _{n\rightarrow \infty }\dfrac{\xi _{k+1}^{(i)}-\xi _k^{(i)}}{\xi _{k+1}^{(1)}+\cdots +\xi _{k+1}^{(i-1)}+\xi _k^{(i)}+\cdots +\xi _k^{(d)}}. \end{aligned}$$

Recall from the proof of Theorem 3.2 that there exists $c_k>0$ and an integrable random variable *W* such that, for any *i*

$$\begin{aligned} \lim _{k\rightarrow \infty }\frac{\xi ^{(i)}_k}{c_k}=W \quad \text {and }\quad \lim _{k\rightarrow \infty }\frac{c_{k+1}}{c_k}=\nu . \end{aligned}$$

Let now $W_1,\ldots ,W_d$ be i.i.d random variables with the same distribution as *W*. It then follows that

$$\begin{aligned} \limsup _{n\rightarrow \infty }\frac{R_n}{n}&\le \alpha +\alpha \limsup _{n\rightarrow \infty }\dfrac{c_k^{-1}\left( \xi _{k+1}^{(i)}-\xi _k^{(i)}\right) }{c_k^{-1}\left( \xi _{k+1}^{(1)}+\cdots +\xi _{k+1}^{(i-1)}+\xi _k^{(i)}+\cdots +\xi _k^{(d)}\right) }\\&=\alpha +\alpha \dfrac{(\nu -1)\max _{i\le d}W_i}{\nu (W_1+\cdots +W_{i-1})+W_i+\cdots +W_d}\\&\le \alpha +\alpha (\nu -1)\frac{\max _{i\le d}W_i}{W_1+\cdots +W_d}. \end{aligned}$$

The same coupling can be applied not only to the *d* children of the root, but also to the vertices of ${\mathbb {T}}_d$ and any given level *j*. This means that, for any $j\ge 0$, we have

$$\begin{aligned} \limsup _{n\rightarrow \infty }\frac{R_n}{n}\le \alpha +\alpha (\nu -1)\frac{\max _{i\le d^j}W_i}{W_1+\cdots +W_{d^j}}. \end{aligned}$$

Since *W* is an integrable random variable, it follows from the law of large numbers that $\frac{\max _{i\le d^j}W_i}{W_1+\cdots +W_{d^j}}\rightarrow 0$ as $j\rightarrow \infty$. Hence, we conclude that

$$\begin{aligned} \limsup _{n\rightarrow \infty }\frac{R_n}{n}\le \alpha . \end{aligned}$$

By symmetry, we can also conclude that $\liminf _{n\rightarrow \infty }\frac{R_n}{n}\ge \alpha$. Theorem 1.1(i) now follows. $\square$

If $\nu$ is the mean offspring number of the supercritical Galton–Watson process $(L_k)$, with $L_k=|\partial _o R_{\tau _k}|$, then we can also write

$$\begin{aligned} \lim _{n\rightarrow \infty }\frac{|R_n|}{n} = \frac{1}{2}\left( 1-\frac{1}{\nu }\right) , \quad \text {almost surely}. \end{aligned}$$

#### Null Recurrent Rotor Walks

In this section, we consider null recurrent rotor walks, that is ${\mathbb {E}}[\rho (v)] = d - 1$. Recall the stopping times $\tau _k$ as defined in (5). The proofs for the law of large numbers for the range will be slightly different, arising from the fact that the expected return time to the sink for the rotor walker is infinite. We first prove the following.

##### Theorem 3.3

For a null recurrent rotor walk $(X_n)$ on ${\mathbb {T}}_d$, $d\ge 2$, we have

$$\begin{aligned} \lim _{k\rightarrow \infty }\dfrac{|R_{\tau _k}|}{\tau _k}=\frac{1}{2}, \text { almost surely}. \end{aligned}$$

##### Proof

Rewriting Eq. (6), we get

$$\begin{aligned} \frac{1}{2}\left( 1-\frac{\tau _{k-1}}{\tau _k}\right) =\frac{|R_{\tau _k}|}{\tau _k},\quad \text {almost surely}, \end{aligned}$$

and we prove that the quotient $\frac{\tau _{k-1}}{\tau _k}$ goes to zero almost surely. We write again ${\mathcal {R}}_k=R_{\tau _k}$ and we first show that $\frac{\tau _k}{\tau _{k-1}}\rightarrow \infty$ almost surely, by finding a lower bound which converges to $\infty$ almost surely. From (6), we have

8 $$\begin{aligned} \frac{\tau _k}{\tau _{k-1}}>2\frac{|{\mathcal {R}}_k|}{\tau _{k-1}},\quad \text {almost surely}. \end{aligned}$$

If $\partial _0 {\mathcal {R}}_{k-1}$ is the set of leaves of ${\mathcal {R}}_{k-1}$, then in the time interval $\tau _k-\tau _{k-1}$, the i.i.d critical Galton–Watson trees rooted at the leaves $\partial _0 {\mathcal {R}}_{k-1}$ will be added to the current range ${\mathcal {R}}_{k-1}$.

Recall that from the proof of Theorem 3.2 that $L_k=|\partial _0 {\mathcal {R}}_k|$. For each $k=1,2,\ldots$, we partition the time interval $(\tau _{k},\tau _{k+1}]$ into finer intervals, on which the behavior of the range can be easily controlled. The vertices in $\partial _oR_{\tau _k}=\{x_1,x_2,\ldots ,x_{L_k}\}$ are ordered from right to left. We introduce the following two (finite) sequences of stopping times $(\eta _k^i)$ and $(\theta _k^i)$ of random length $L_k+1$, as follows: let $\theta _k^0=\tau _k$ and $\eta _k^{L_k+1}=\tau _{k+1}$ and for $i=1,2,\ldots ,L_k$

9 $$\begin{aligned} \begin{aligned} \eta _k^i=&\min \{j> \theta _k^{i-1}:\ X_j=x_i\} \\ \theta _k^i=&\min \{j>\eta _k^i:\ X_j=x_i\text { and } \rho (x_j)=x_i^{(d)}\}. \end{aligned} \end{aligned}$$

That is, for each leaf $x_i$, the time $\eta _k^i$ represents the first time the rotor walk reaches $x_i$, and $\theta _k^i$ represents the last time the rotor walk returns to $x_i$ after making a full excursion in the critical Galton–Watson tree rooted at $x_i$. Then

$$\begin{aligned} (\tau _k,\tau _{k+1}]=\left\{ \cup _{i=1}^{L_k+1}\left( \theta _k^{i-1},\eta _k^i\right] \right\} \cup \left\{ \cup _{i=1}^{L_k}\left( \eta _k^i,\theta _k^i\right] \right\} , \end{aligned}$$

almost surely. It is easy to see that the increments $(\theta _k^i-\eta _k^i)_{i}$ are i.i.d and distributed according to the distribution of $\tau _1$, which is the time a rotor walk needs to return to the sink for the first time. Once the rotor walk reaches the leaf $x_i$ for the first time at time $\eta _k^i$, the subtree rooted at $x_i$ was never visited before by a rotor walk. Even more, the tree of good children with root $x_i$ is a critical Galton–Watson tree, which becomes extinct almost surely. Thus, the rotor walk on this subtree is (null) recurrent, and it returns to $x_i$ at time $\theta _k^i$. Then $(\theta _k^i-\eta _k^i)$ represents the length of this excursion which has expectation ${\mathbb {E}}[\tau _1]=\infty$. In the time interval $(\theta _k^{i-1},\eta _k^i]$, the rotor walk leaves the leaf $x_{i-1}$ and returns to the confluent between $x_{i-1}$ and $x_i$, from where it continues its journey until it reaches $x_i$. Then $\eta _k^i-\theta _k^{i-1}$ is the time the rotor walk needs to reach the new leaf $x_i$ after leaving $x_{i-1}$. In this time interval, the range does not change, since $(X_n)$ makes steps only in $R_{\tau _k}$. We have, as a consequence of (6)

10 $$\begin{aligned} |{\mathcal {R}}_k|=|{\mathcal {R}}_{k-1}|+\frac{1}{2}\sum _{i=1}^{L_{k-1}}\left( \theta _{k-1}^i-\eta _{k-1}^i\right) ,\quad \text { almost surely}. \end{aligned}$$

By the strong law of large numbers, we have on the one side

11 $$\begin{aligned} \frac{\sum _{i=1}^{L_{k-1}}\left( \theta _{k-1}^i-\eta _{k-1}^i\right) }{L_{k-1}}\rightarrow {\mathbb {E}}[\tau _1]=\infty ,\quad \text {almost surely}. \end{aligned}$$

From (2) we obtain $L_{k-1}=1+(d-1)|{\mathcal {R}}_{k-1}|$ which together with (8) and (10) yields

12 $$\begin{aligned} \frac{\tau _k}{\tau _{k-1}}&>\frac{\sum _{i=1}^{L_{k-1}}\left( \theta _{k-1}^i-\eta _{k-1}^i\right) }{L_{k-1}}\cdot \frac{1+(d-1)|{\mathcal {R}}_{k-1}|}{\tau _{k-1}} \end{aligned}$$

13 $$\begin{aligned}&\ge \frac{\sum _{i=1}^{L_{k-1}}\left( \theta _{k-1}^i-\eta _{k-1}^i\right) }{L_{k-1}}\cdot \frac{|{\mathcal {R}}_{k-1}|}{\tau _{k-1}} \end{aligned}$$

14 $$\begin{aligned}&= \frac{\sum _{i=1}^{L_{k-1}}\left( \theta _{k-1}^i-\eta _{k-1}^i\right) }{L_{k-1}}\cdot \frac{1}{2}\left( 1-\frac{\tau _{k-2}}{\tau _{k-1}}\right) , \end{aligned}$$

almost surely, where the last inequality follows from (6). By letting $l=\liminf \frac{\tau _k}{\tau _{k-1}}$ and taking limits, the previous equation yields

$$\begin{aligned} l\ge \frac{1}{2}{\mathbb {E}}[\tau _1]\left( 1-\frac{1}{l}\right) . \end{aligned}$$

Unless $l=1$, the right-hand side above goes to infinity almost surely, which implies $l=\infty =\liminf \frac{\tau _k}{\tau _{k-1}}\le \limsup \frac{\tau _k}{\tau _{k-1}}$, therefore $\lim _k \frac{\tau _k}{\tau _{k-1}}=\infty$ and $\lim _k \frac{\tau _{k-1}}{\tau _{k}}=0$, almost surely. Suppose now $l=1$, almost surely. Once at the root at time $\tau _{k-1}$, until the next return at time $\tau _k$, the rotor walk visits everything that was visited before plus new trees where the configuration is in the initial status. As a consequence of Lemma 3.1, for visiting the previously visited set, it needs time $\tau _{k-1}$, therefore $\tau _k-\tau _{k-1}>\tau _{k-1}$, which gives that $\liminf _k\frac{\tau _k}{\tau _{k-1}}>2$, which contradicts the fact that $l=1$. Therefore $\lim _k \frac{\tau _k}{\tau _{k-1}}=\infty$, almost surely. Finally, we show that indeed $\lim _{k\rightarrow \infty }\frac{|{\mathcal {R}}_k|}{\tau _k}$ exists. On the one hand, from (6), it is easy to see that $\frac{|{\mathcal {R}}_k|}{\tau _k}\le \frac{1}{2}$. We have

$$\begin{aligned} \frac{1}{2}\ge \frac{|{\mathcal {R}}_k|}{\tau _k} \ge \liminf \frac{|{\mathcal {R}}_k|}{\tau _k}=\frac{1}{2}\liminf \left( 1-\frac{\tau _{k-1}}{\tau _k}\right) =\frac{1}{2}, \end{aligned}$$

almost surely, and the claim follows. $\square$

##### Proof of Theorem 1.1(ii)

Set again ${\mathcal {R}}_k:=R_{\tau _k}$ and recall from the proof of Theorem 3.3, the definition of the stopping times $\eta _k^i$ and $\theta _k^i$, for $i=1,\ldots ,L_k=|\partial _0{\mathcal {R}}_k|$. Let *n* be an arbitrary positive number in $(\tau _k,\tau _{k+1}]$. Then there exists $i\in \{1,\ldots ,L_k\}$ such that $n\in (\theta _k^{i-1},\theta _k^i].$ Then we have

15 $$\begin{aligned} |{\mathcal {R}}_k|+\sum _{j=1}^{i-1}|{\mathcal {R}}_1^j|&\le |R_n|\le |{\mathcal {R}}_k|+\sum _{j=1}^{i}|{\mathcal {R}}_1^j| \end{aligned}$$

16 $$\begin{aligned} \tau _k+\sum _{j=1}^{i-1}\tau _1^j&\le n \le \tau _k+\sum _{j=1}^{i}\tau _1^j, \end{aligned}$$

where $({\mathcal {R}}_1^j)_j$ and $(\tau _1^j)_j$ are i.i.d. random variables distributed like ${\mathcal {R}}_{1}=R_{\tau _1}$ and $\tau _1$, respectively. From (6) we have $\tau _1^j=2|{\mathcal {R}}_1^j|$, and from the previous two equations we obtain the following upper bound on $\frac{|R_n|}{n}$:

$$\begin{aligned} \frac{|R_n|}{n}&\le \frac{1}{2}\left[ \dfrac{\tau _k-\tau _{k-1}+\sum _{j=1}^i \tau _1^j}{\tau _k+\sum _{j=1}^{i-1} \tau _1^j} \right] =\frac{1}{2}\left[ 1+\dfrac{\tau _1^i}{\tau _k+\sum _{j=1}^{i-1} \tau _1^j}-\dfrac{\tau _{k-1}}{\tau _k+\sum _{j=1}^{i-1} \tau _1^j}\right] . \end{aligned}$$

Since for every *i*, $\tau _1^i$ is almost surely finite and $\tau _k\rightarrow \infty$ as $k\rightarrow \infty$, the term $\dfrac{\tau _1^i}{\tau _k+\sum _{j=1}^{i-1} \tau _1^j}$ converges almost surely to 0 as $k\rightarrow \infty$. Moreover, since in the null recurrent case, from the proof of Theorem 3.3, $\frac{\tau _{k-1}}{\tau _k}\rightarrow 0$ almost surely, as $k\rightarrow \infty$, we also get the almost sure convergence to 0 of $\dfrac{\tau _{k-1}}{\tau _k+\sum _{j=1}^{i-1} \tau _1^j}$. Taking limits on both sides in the equation above, we obtain that

$$\begin{aligned} \limsup _{n\rightarrow \infty }\frac{|R_n|}{n}\le \frac{1}{2},\quad \text {almost surely}. \end{aligned}$$

For the lower bound, Eqs. (15) and (16) yield

$$\begin{aligned} \frac{|R_n|}{n}&\ge \frac{1}{2}\left[ \dfrac{\tau _k-\tau _{k-1}+\sum _{j=1}^{i-1} \tau _1^j}{\tau _k+\sum _{j=1}^{i} \tau _1^j} \right] = \frac{1}{2}\left[ 1-\dfrac{\tau _1^i}{\tau _k+\sum _{j=1}^{i} \tau _1^j}-\dfrac{\tau _{k-1}}{\tau _k+\sum _{j=1}^{i} \tau _1^j}\right] , \end{aligned}$$

and by the same reasoning as above, we obtain

$$\begin{aligned} \liminf _{n\rightarrow \infty }\frac{|R_n|}{n}\ge \frac{1}{2},\quad \text {almost surely}, \end{aligned}$$

which together with the upper bound on limsup proves that $\lim _{n\rightarrow \infty }\frac{|R_n|}{n}=\frac{1}{2}$ almost surely. $\square$

### Transient Rotor Walks

We consider here the transient case on regular trees, when ${\mathbb {E}}[\rho (v)]<d-1$. Then each vertex is visited only finitely many times, and the walk escapes to infinity along a live path. The tree ${\mathcal {T}}^{\mathsf {good}}_d$ of good children for $(X_n)$ is a supercritical Galton–Watson tree, with mean offspring number $m=d-{\mathbb {E}}[\rho (v)]>1$. Thus, ${\mathcal {T}}^{\mathsf {good}}_d$ survives with positive probability $(1-q)$, where $q\in (0,\infty )$ is the extinction probability, and is the smallest nonnegative root of the equation $f(s)=s$, where *f* is the generating function of ${\mathcal {T}}^{\mathsf {good}}_d$.

#### Notation 3.4

*For the rest of this section, we will always condition on the event of non-extinction, so that*${\mathcal {T}}^{\mathsf {good}}_d$*is an infinite random tree.* We denote by ${\mathbb {P}}_{\mathsf {non}}$ and by ${\mathbb {E}}_{\mathsf {non}}$ the associated probability and expectation conditioned on non-extinction, respectively.

That is, if ${\mathbb {P}}$ is the probability for the rotor walk in the original tree ${\mathcal {T}}^{\mathsf {good}}_d$, then for some event *A*, we have

$$\begin{aligned} {\mathbb {P}}_{\mathsf {non}}[A]=\frac{{\mathbb {P}}[A\cap {\mathcal {T}}^{\mathsf {good}}_d \text { is infinite}]}{1-q}. \end{aligned}$$

In order to understand how the rotor walk $(X_n)$ escapes to infinity, we will decompose the tree ${\mathcal {T}}^{\mathsf {good}}_d$ with generating function *f* conditioned on non-extinction. Consider the generating functions

17 $$\begin{aligned} g(s)=\dfrac{f\left( (1-q)s+q\right) -q}{1-q} \quad \text {and} \quad h(s)=\dfrac{f(qs)}{q}. \end{aligned}$$

Then the *f*-Galton–Watson tree ${\mathcal {T}}^{\mathsf {good}}_d$ can be generated by:

(i) growing a Galton–Watson tree ${\mathcal {T}}_g$ with generating function *g*, which has the survival probability 1.
(ii) attaching to each vertex *v* of ${\mathcal {T}}_g$ a random number $n_v$ of h-Galton–Watson trees, acting as traps in the environment ${\mathcal {T}}^{\mathsf {good}}_d$.

The tree ${\mathcal {T}}_g$ is equivalent (in the sense of finite dimensional distributions) with a tree in which all vertices have an infinite line of descent. ${\mathcal {T}}_g$ is called the *backbone* of ${\mathcal {T}}^{\mathsf {good}}_d$. The random variable $n_v$ has a distribution depending only on *d*(*v*) in ${\mathcal {T}}^{\mathsf {good}}_d$ and given ${\mathcal {T}}_g$ and $n_v$ the traps are i.i.d. The supercritical Galton–Watson tree ${\mathcal {T}}^{\mathsf {good}}_d$ conditioned to die out is equivalent to the *h*-Galton–Watson tree with generating function *h*, which is subcritical. For more details on this decomposition and the equivalence of the processes involved above, see [9] and [2, Chapter I, Part D].

The exposition in this paragraph is a non-trivial consequence of the key Lemma 3.1(b). Denote by $t_0<\infty$ the number of times the origin was visited. After the $t_0$-th visit to the origin, there are no returns to the origin, almost surely, and there has to be a leaf $\gamma _0$ belonging to the range of the rotor walk up to the $t_0$-th return, along which the rotor walker escapes to infinity, that is, there is a live path starting at $\gamma _0$, almost surely. Denote by $n_0$ the first time the rotor walk arrives at $\gamma _0$. The tree rooted at $\gamma _0$ was not visited previously by the walker, and the rotors are in their random initial configuration. The tree of good children ${\mathcal {T}}^{\mathsf {good}}_d$ rooted at $\gamma _0$ in the initial rotor configuration has the same distribution as the supercritical Galton–Watson tree conditioned on non-extinction. At time $n_0$, we have already a finite visited subtree and its cardinality $|R_{n_0}|$, which is negligible for the limit behavior of the range. When computing the limit for the size of the range, we have to consider also this irrelevant finite part.

On the event of non-extinction, let $\gamma =(\gamma _0,\gamma _1,\ldots )$ be the rightmost infinite ray in ${\mathcal {T}}^{\mathsf {good}}_d$ rooted at $\gamma _0$, that is, the rightmost infinite live path in ${\mathbb {T}}_d$, which starts at $\gamma _0$. This is the rightmost ray in the tree ${\mathcal {T}}_g$. Since all vertices in ${\mathcal {T}}_g$ have an infinite line of descent, such a ray exists. The ray $\gamma$ is then a live path, along which the rotor walk $(X_n)$ escapes to infinity, without visiting the vertices to the left of $\gamma$; see again Lemma 3.1(b). In order to understand the behavior of the range of $(X_n)$ and to prove a law of large numbers, we introduce the sequence of *regeneration times*$(\tau _k)$ for the ray $\gamma$. Let $\tau _0=n_0$ and for $k\ge 1$:

18 $$\begin{aligned} \tau _k=\inf \{n\ge n_0: X_n=\gamma _k\}. \end{aligned}$$

Note that, for each *k* the random times $\tau _k$ and $\tau _{k+1}-1$ are the first and the last hitting time of $\gamma _k$, respectively. Indeed, once we are at vertex $\gamma _k$, since $\gamma _{k+1}$ is the rightmost child of $\gamma _k$ with infinite line of descent, the rotor walk visits all good children to the right of $\gamma _k$, and makes finite excursions in the trees rooted at those good children, and then returns to $\gamma _k$ at time $\tau _{k+1}-1$. Then, at time $\tau _{k+1}$, the walk moves to $\gamma _{k+1}$ and never returns to $\gamma _k$. We first prove the following.

#### Theorem 3.5

Let $(X_n)$ be a transient rotor walk on ${\mathbb {T}}_d$. If the tree ${\mathcal {T}}^{\mathsf {good}}_d$ of good children for the rotor walk $(X_n)$ has extinction probability *q*, then conditioned on non-extinction, there exists a constant $\alpha >0$, which depends only on *q* and *d*, such that

$$\begin{aligned} \lim _{k\rightarrow \infty }\frac{|R_{\tau _k}|}{\tau _k}=\alpha ,\quad \text {almost surely}, \end{aligned}$$

and $\alpha$ is given by

$$\begin{aligned} \alpha =\dfrac{q-f'(q)(q^2-q+1)}{q^2+q-f'(q)(2q^2-q+1)}. \end{aligned}$$

#### Proof

We write again ${\mathcal {R}}_k:=R_{\tau _k}$, and ${\mathcal {R}}_0$ for the range of the rotor walk up to time $n_0$, which is finite almost surely. Since $\gamma$ is the rightmost infinite live path on which $(X_n)$ escapes to infinity, to the right of each vertex $\gamma _k$ in ${\mathcal {T}}^{\mathsf {good}}_d$ we have a random number of vertices, and in the tree rooted at those vertices (which are *h*-Galton–Watson trees), the rotor walk makes only finite excursions. Then, at time $\tau _{k+1}$, the walk reaches $\gamma _{k+1}$ and never returns to $\gamma _k$.

For each *k*, $\gamma _{k+1}$ is a good child of $\gamma _k$ and the rotor at $\gamma _k$ points to the right of $\gamma _{k+1}$.

For $k=0,1,\ldots$ let $\left\{ \gamma _k^{(1)},\ldots ,\gamma _k^{(N_k)}\right\}$ be the set of vertices which are good children of $\gamma _k$, and are situated to the right of $\gamma _{k+1}$; denote by $N_k$ the cardinality of this set. Additionally, denote by ${\widetilde{T}}_k(j)$ the tree rooted at $\gamma _k^{(j)}$, $j=1,\ldots , N_k$ and by $T_k(j)={\widetilde{T}}_k(j)\cup (\gamma _k,\gamma _k^{(j)})$. That is, the trees $T_k(j)$, for $j=1,\ldots , N_k$ have all common root $\gamma _k$, and $|T_k(j)|=|{\widetilde{T}}_k(j)|+1$.

**Claim 1.***Conditionally on the event of non-extinction*, $(N_k)_{k\ge 0}$*are i.i.d*.

*Proof of Claim 1. * For each *k*, the distribution of $N_k$ depends only on the offspring distribution (which is the number of good children for the rotor walk) of ${\mathcal {T}}_d^{\mathsf {good}}$, and the last one depends only on the initial rotor configuration $\rho$. Since the random variables $(\rho (v))_v$ are i.i.d, the claim follows.

**Claim 2.***Conditionally on the event of non-extinction*, $(|T_k(j)|)_{1\le j\le N_k}$*are i.i.d*.

*Proof of Claim 2. * This follows immediately from the definition of the Galton–Watson tree, since each vertex in ${\mathcal {T}}_d^{\mathsf {good}}$ has *k* children with probability $r_{d-k}$, independently of all other vertices, and all these children have independent Galton–Watson descendant subtrees. All the subtrees ${\widetilde{T}}_k(j)$ rooted at $\gamma _k^{(j)}$ are then independent h-Galton–Watson subtrees (subcritical), therefore $(|{\widetilde{T}}_k(j)|)_{1\le j\le N_k}$ are i.i.d. Since $|T_k(j)|=|{\widetilde{T}}_k(j)|+1$, the claim follows.

**Claim 3.***Given non-extinction, the increments*$(\tau _{k+1}-\tau _k)_{k\ge 0}$*are i.i.d.*

*Proof of Claim 3. * Given non-extinction, the time $(\tau _{k+1}-\tau _k)_{k\ge 0}$ depends only on $N_k$ (the number of good children to the right of $\gamma _k$) and on $|T_k(j)|$, which by Claims 1 and 2 above, are all i.i.d. Thus, the independence of $(\tau _{k+1}-\tau _k)_{k\ge 0}$ follows as well.

Clearly, each $\gamma _k$ is visited exactly $N_{k}+1$ times, and all vertices to the left of $\gamma$ are never visited. We write ${\mathcal {R}}_{(k-1,k]}$ for the range of the rotor walk in the time interval $(\tau _{k-1},\tau _k]$. For $r\ne s$, the path of the rotor walk in the time interval $(\tau _{r-1},\tau _r]$ has empty intersection with the path in the time interval $(\tau _{s-1},\tau _s]$, and we have

$$\begin{aligned} |{\mathcal {R}}_k|=|{\mathcal {R}}_0|+\sum _{i=1}^k|{\mathcal {R}}_{(i-1,i]}|,\quad \text {almost surely}. \end{aligned}$$

Moreover, for $$i=1,\ldots k$$,

$$\begin{aligned} |{\mathcal {R}}_{(i-1,i]}|=1+\sum _{j=1}^{N_{i-1}}|{\widetilde{T}}_{i-1}(j)|,\quad \text {almost surely} \end{aligned}$$

and if we denote by $\alpha _k=\sum _{i=0}^{k-1}N_i$ and if $({{\tilde{t}}}_j)$ is an i.i.d. sequence of random variables with the same distribution as $|{\widetilde{T}}_{i-1}(j)|$, then, using Claim 1 and Claim 2 we obtain

19 $$\begin{aligned} |{\mathcal {R}}_k|=|{\mathcal {R}}_0|+k+\sum _{j=1}^{\alpha _k}{{\tilde{t}}}_j,\quad \text { almost surely}. \end{aligned}$$

Similarly, if we write $\tau _k=n_0+\sum _{i=2}^k(\tau _i-\tau _{i-1})$ and use the fact that, for $i\ge 2$ as a consequence of (6)

$$\begin{aligned} \tau _i-\tau _{i-1}=1+2\sum _{j=1}^{N_{i-1}}|{\widetilde{T}}_{i-1}(j)|, \quad \text {almost surely}, \end{aligned}$$

then again by Claims 1 and 2 we get

20 $$\begin{aligned} \tau _k=|{\mathcal {R}}_0|+k+2\sum _{j=1}^{\alpha _k}{\tilde{t}}_j. \end{aligned}$$

Putting Eqs. (19) and (20) together, we finally get

$$\begin{aligned} \frac{|{\mathcal {R}}_k|}{\tau _k}=\dfrac{|{\mathcal {R}}_0|+k+\sum _{j=1}^{\alpha _k}{\tilde{t}}_j}{|{\mathcal {R}}_0|+k+2\sum _{j=1}^{\alpha _k}{\tilde{t}}_j},\quad \text {almost surely}. \end{aligned}$$

By the strong law of large numbers, we have

$$\begin{aligned} \frac{|{\mathcal {R}}_0|+k}{\alpha _k}\rightarrow \frac{1}{{\mathbb {E}}_{\mathsf {non}}[N_0]},\quad \frac{\sum _{j=1}^{\alpha _k}{\tilde{t}}_j}{\alpha _k}\rightarrow {\mathbb {E}}_{\mathsf {non}}[|{\widetilde{T}}_0(1)|]\quad \text {almost surely} \end{aligned}$$

which implies

$$\begin{aligned} \frac{|{\mathcal {R}}_k|}{\tau _k}\rightarrow \dfrac{1+{\mathbb {E}}_{\mathsf {non}}[N_0]{\mathbb {E}}_{\mathsf {non}}[|{\widetilde{T}}_0(1)|]}{1+2{\mathbb {E}}_{\mathsf {non}}[N_0]{\mathbb {E}}_{\mathsf {non}}[|{\widetilde{T}}_0(1)|]},\quad \text {almost surely}. \end{aligned}$$

We have to compute now the two expectations ${\mathbb {E}}_{\mathsf {non}}[N_0]$ and ${\mathbb {E}}_{\mathsf {non}}[|{\widetilde{T}}_0(1)|]$ involved in the equation above, in order to get the formula from the statement of the theorem.

Conditioned on non-extinction, for all $k=0,1,\ldots$ and $j=1,\ldots N_k$ the trees ${\widetilde{T}}_k(j)$ are i.i.d. subcritical Galton–Watson trees with generating function $h(s)=\frac{f(qs)}{q}$ and mean offspring number $h'(1)$. That is, they all die out with probability one, and the expected number of vertices is ${\mathbb {E}}_{\mathsf {non}}[|{\widetilde{T}}_k(j)|]=\frac{1}{1-h'(1)}$. On the other hand, $h(s)=\frac{1}{q}\sum _{j=0}^d r_{d-j}(qs)^j$, which implies that $h'(1)=\frac{1}{q^2}\sum _{j=0}^d jr_{d-j} q^j$, and

$$\begin{aligned} {\mathbb {E}}_{\mathsf {non}}[{\widetilde{T}}_0(1)]=\dfrac{q^2}{q^2-\sum _{j=0}^d jr_{d-j} q^j}, \end{aligned}$$

which in terms of the generating function *f*(*s*) can be written as

21 $$\begin{aligned} {\mathbb {E}}_{\mathsf {non}}[{\widetilde{T}}_0(1)]=\frac{q}{q-f'(q)}. \end{aligned}$$

In computing $${\mathbb {E}}_{\mathsf {non}}[N_0]$$:

$$\begin{aligned}&{\mathbb {E}}_{\mathsf {non}}[N_0]=\sum _{i=0}^{d-1}i{\mathbb {P}}_{\mathsf {non}}[N_0=i]=\frac{1}{1-q}\sum _{i=0}^{d-1}i{\mathbb {P}}[N_0=i, {\mathcal {T}}_d^{\mathsf {good}} \text { is infinite}]\\&\quad =\frac{1}{1-q}\sum _{j=0}^d\sum _{i=0}^{j-1}i {\mathbb {P}}[N_0=i,\ {\mathcal {T}}_d^{\mathsf {good}} \text { is infinite}|\gamma _0 \text { has j good children}]\\&\qquad {\mathbb {P}}[\gamma _0 \text { has j good children}]\\&\quad =\frac{1}{1-q}\sum _{j=0}^{d}r_{d-j}\sum _{i=0}^{j-1}iq^i(1-q)=\sum _{j=0}^{d}r_{d-j}\sum _{i=0}^{j-1}iq^i)\\&\quad =\sum _{j=0}^{d}r_{d-j}\frac{(j-1)q^{j+1}-jq^j+q}{(1-q)^2}\\&\quad =\frac{q}{(1-q)^2}\left( 1-\sum _{j=0}^dr_{d-j}q^j-\frac{1-q}{q}\sum _{j=0}^d jr_{d-j}q^j\right) \\&\quad =\frac{q}{1-q}\left( 1-\sum _{j=1}^d jr_{d-j}q^{j-1}\right) \end{aligned}$$

In terms of the generating function *f*(*s*), using $f(q)=q$, ${\mathbb {E}}_{\mathsf {non}}[N_0]$ can be written as

22 $$\begin{aligned} {\mathbb {E}}_{{\mathsf {non}}}[N_0]=\frac{q}{1-q}\left( 1-f'(q)\right) . \end{aligned}$$

Putting the two expectations together, we obtain the constant $\alpha$ in terms of the generating function *f*(*s*) given by

$$\begin{aligned} \alpha =\dfrac{q-f'(q)(q^2-q+1)}{q^2+q-f'(q)(2q^2-q+1)}=\dfrac{q-(q^2-q+1)\sum _{j=1}^d jr_{d-j}q^{j-1}}{q^2+q-(2q^2-q+1)\sum _{j=1}^d jr_{d-j}q^{j-1}}. \end{aligned}$$

$$\square $$

In Theorem 3.5, we have proved a law of large numbers for the range of the rotor walk along a subsequence $(\tau _k)$. With very little effort, we can show that we have indeed a law of large numbers at all times.

#### Proof of Theorem 1.1(iii)

For $n\in {\mathbb {N}}$, the infinite ray $\gamma$ and the regeneration times $(\tau _k)$ as defined in (18) let

$$\begin{aligned} k=\max \{j: \tau _j< n\}. \end{aligned}$$

Then $\tau _k< n\le \tau _{k+1}$ a.s. and $|R_{\tau _k}|\le |R_n| \le |R_{\tau _{k+1}} |$ a.s. which in turn gives

23 $$\begin{aligned} \frac{\tau _{k+1}}{\tau _k} \cdot \frac{|R_{\tau _k}|}{\tau _k}\le \frac{|R_n|}{n}\le \frac{|R_{\tau _{k+1}}|}{\tau _{k+1}}\cdot \frac{\tau _k}{\tau _{k+1}},\quad \text {almost surely}. \end{aligned}$$

By Claim 3 from the proof of Theorem 3.5, the increments $(\tau _{k+1}-\tau _k)$ are i.i.d, and finite almost surely, therefore $\frac{\tau _{k+1}-\tau _k}{\tau _k}\rightarrow 0$ almost surely as $k\rightarrow \infty$. Then, since

$$\begin{aligned} \frac{\tau _{k+1}}{\tau _k}=\frac{\tau _{k+1}-\tau _k}{\tau _k}+1, \end{aligned}$$

we have that $\frac{\tau _{k+1}}{\tau _k}\rightarrow 1$ almost surely, as $k\rightarrow \infty$. This, together with Theorem 3.5 implies that the left-hand side of Eq. (23) converges to $\alpha$ almost surely. By the same argument, we obtain that also the right-hand side of (23) converges to the same constant $\alpha$ almost surely, and this completes the proof. $\square$

### Uniform Rotor Walks

We discuss here the behavior of rotor walks on regular trees ${\mathbb {T}}_d$, with uniform initial rotor configuration $\rho$, that is, for all $v\in {\mathbb {T}}_d$, the random variables $(\rho (v))$ are i.i.d with uniform distribution on the set $\{0,1,\ldots ,d\}$, i.e., ${\mathbb {P}}[\rho (v)=j]=\frac{1}{d+1}$, for $j\in \{0,1,\ldots ,d\}$. Such walks are null recurrent on ${\mathbb {T}}_2$ and transient on all ${\mathbb {T}}_d$, $d\ge 3$. As a special case of Theorem 1.1(iii), we have the following.

#### Corollary 3.6

If $(X_n)$ is a uniform rotor walk on ${\mathbb {T}}_d$, $d\ge 3$, then the constant $\alpha$ is given by

$$\begin{aligned} \alpha =\dfrac{q(1-d)(1-q^{d+1})}{(d+1)(q-1)^3}+\dfrac{q^2(1-q^{d-1})}{(q-1)^3}. \end{aligned}$$

#### Proof

Using that ${\mathbb {P}}[\rho (v)=j]=\frac{1}{d+1}=r_{d-j}$ and putting $f'(q)=\frac{q^d}{q-1}-\frac{q^{d+1}-1}{(d+1)(q-1)^2}$ in Theorem 3.5, we get the result. $\square$

The following table shows values for the constants $\alpha$ in comparison with the limit $(d-1)/d$ for the simple random walk on trees.

**Table:** 

*d*	$\alpha$	$(d-1)/d$
2	0.500	0.500
3	0.707	0.666
4	0.784	0.750
5	0.825	0.800
6	0.853	0.833
7	0.872	0.857
8	0.888	0.875
9	0.899	0.888
10	0.909	0.900

Note that only in the case of the binary tree ${\mathbb {T}}_2$, the limit values for the range of the uniform rotor walk and of the simple random walk are equal, even though the uniform rotor walk is null recurrent and the simple random walk is transient. In the transient case, that is, for all $d\ge 3$ we always have $\alpha > (d-1)/d$.

## Speed on Regular Trees

In this section, we prove the existence of the almost sure limit $\frac{|X_n|}{n}$, as $n\rightarrow \infty$, where $|X_n|$ represents the distance from the root to the position $X_n$ at time *n* of the walker.

### Recurrent Rotor Walks

#### Proof of Theorem 1.2(i)

We show that in this case, $l=\lim _{n\rightarrow \infty }\frac{|X_n|}{n}=0$, almost surely. For arbitrary *n*, let

$$\begin{aligned} k=\max \{ i: \tau _i< n\}, \end{aligned}$$

which implies that $\tau _k< n\le \tau _{k+1}$, and up to time *n* we have *k* returns to the root. Let $D_k$ be the maximum distance from the root reached after *k* returns to the root.

*Positive recurrent rotor walks.* We have

$$\begin{aligned} 0\le \frac{|X_n|}{n}\le \frac{D_{k+1}}{\tau _k}=\frac{D_{k+1}}{k+1}\cdot \frac{k+1}{\tau _k}. \end{aligned}$$

In view of [1, Theorem 7(ii)], the maximal depth grows linearly with the number of returns to the root, that is, $\frac{D_{k+1}}{k+1}$ is almost surely bounded. On the other hand, $\tau _k$ grows exponentially in *k*, therefore $\frac{k+1}{\tau _k}$ converges almost surely to 0 as $k\rightarrow \infty$, that is $l=\lim _{n\rightarrow \infty }\frac{|X_n|}{n}=0$ almost surely.

*Null recurrent rotor walks.* In the null recurrent case, the situation is a bit different, since even though all particles return to the root, they reach very great depths; see again [1, Theorem 7(i)]. Write again ${\mathcal {R}}_k:=R_{\tau _k}$. For the position of the rotor walk $X_n$, we distinguish the following three cases:

(i) If $$X_n\in {\mathcal {R}}_{k-1}$$, then
  $$\begin{aligned} 0\le \frac{|X_n|}{n}\le \frac{| {\mathcal {R}}_{k-1}|}{\tau _k}=\frac{| {\mathcal {R}}_{k-1}|}{\tau _{k-1}}\cdot \frac{\tau _{k-1}}{\tau _k}, \end{aligned}$$
  and the right-hand side above converges to 0 almost surely in view of Theorem 3.3 together with the fact $\frac{\tau _{k-1}}{\tau _k}\rightarrow 0$ almost surely, proven again in Theorem 3.3. Therefore $\lim _{n\rightarrow \infty }\frac{|X_n|}{n}=0$ almost surely.
(ii) If $X_n\in {\mathcal {R}}_{k}{\setminus }{\mathcal {R}}_{k-1}$, then there exists $i\in \{1,2,\ldots ,L_{k-1}\}$, where $L_{k-1}=|\partial _0{\mathcal {R}}_{k-1}|$, such that $X_n$ is in the tree rooted at $x_i$, and
  $$\begin{aligned} 0\le \frac{|X_n|}{n}\le \frac{| {\mathcal {R}}_{k-1}|}{\tau _k}+\frac{\tau _1^i}{\tau _k}, \end{aligned}$$
  where $\tau _1^i$ is a random variable, independent and identically distributed to $\tau _1$ which is finite almost surely. The quantity $\frac{| {\mathcal {R}}_{k-1}|}{\tau _k}$ converges to 0 by the same argument as in case (i), whereas $\frac{\tau _1^i}{\tau _k}$ converges also to 0 almost surely, as $k=k(n)\rightarrow \infty .$
(iii) Finally, if $X_n\in {\mathcal {R}}_{k+1}{\setminus } {\mathcal {R}}_{k}$, then there exist $i\in \{1,2,\ldots ,L_{k-1}\}$ and $j\in \{1,2,\ldots ,L_{k}\}$ such that
  $$\begin{aligned} 0\le \frac{|X_n|}{n}\le \frac{| {\mathcal {R}}_{k-1}|}{\tau _k}+\frac{\tau _1^i}{\tau _k}+\frac{\tau _1^j}{\tau _k}, \end{aligned}$$
  where both $\tau _1^i,\tau _1^j$ are i.i.d random variables, identically distributed as $\tau _1$ which is finite almost surely, and the right-hand side converges again to 0 almost surely, as $k=k(n)\rightarrow \infty$, and this proves the claim. $\square$

### Transient Rotor Walks

Since in the transient case there is a positive probability of extinction of ${\mathcal {T}}_{d}^{\mathsf {good}}$, we condition on the event of non-extinction. The notation remains the same as in Sect. 3.2.

#### Proof of Theorem 1.2(ii)

Recall the definition of the infinite ray $\gamma$ along which the rotor walk $(X_n)$ escapes to infinity, and the regeneration times $\tau _k$ as defined in (18). We first prove the existence of the speed *l* along the sequence $(\tau _k)$. This is rather easy, since $d(r,\gamma _k)=|X_{n_0}|+k$, a.s. where $n_0<\infty$ is the first time the walk reaches $\gamma _0$, from where it escapes without returning to the root; see again Lemma 3.1(b).

Recall now from the proof of Theorem 3.5, that $\tau _k=n_0+k+2\sum _{j=1}^{\alpha _k}{\tilde{t}}_j$ a.s. and $\alpha _k=\sum _{i=0}^{k-1}N_i$ a.s., with the involved quantities again as computed in Theorem 3.5. Then

$$\begin{aligned} \frac{|X_{\tau _k}|}{\tau _k}=\frac{|X_{n_0}|+k}{\tau _k}=\frac{1+\frac{|X_{n_0}|}{k}}{1+\frac{n_0}{k}+\frac{2\sum _{j=1}^{\alpha _k}{\tilde{t}}_j}{\alpha _k}\cdot \frac{\alpha _k}{k}}. \end{aligned}$$

By the strong law of large numbers for sums of i.i.d random variables, we have $\frac{2\sum _{j=1}^{\alpha _k}{\tilde{t}}_j}{\alpha _k}\rightarrow {\mathbb {E}}_{\mathsf {non}}[|{\widetilde{T}}_0(1)|]$ and $\frac{\alpha _k}{k}\rightarrow {\mathbb {E}}_{\mathsf {non}}[N_0]$ almost surely, as $k\rightarrow \infty$, Also, since $|X_{n_0}|$ and $n_0$ are both finite, the almost sure limits $\frac{|X_{n_0}|}{k}$ and $\frac{n_0}{k}$ are 0, as $k\rightarrow \infty$. This implies

$$\begin{aligned} \frac{|X_{\tau _k}|}{\tau _k}\rightarrow \frac{1}{1+2{\mathbb {E}}_{\mathsf {non}}[|{\widetilde{T}}_0(1)|]{\mathbb {E}}_{\mathsf {non}}[N_0]},\quad \text {almost surely, as } k\rightarrow \infty . \end{aligned}$$

By Eqs. (21) and (22), which give ${\mathbb {E}}_{\mathsf {non}}[|{\widetilde{T}}_0(1)|]$ and ${\mathbb {E}}_{\mathsf {non}}[N_0]$ in terms of the generating function *f* of the tree ${\mathcal {T}}^{\mathsf {good}}_d$, we obtain

24 $$\begin{aligned} \frac{|X_{\tau _k}|}{\tau _k}\rightarrow \frac{(q-f'(q))(1-q)}{q+q^2-f'(q)(2q^2-q+1)}=l,\quad \text { almost surely as }k\rightarrow \infty . \end{aligned}$$

In order to prove the almost sure convergence of $\frac{|X_n|}{n}$, for all *n*, we take

$$\begin{aligned} k=\max \{ i: \tau _i< n\}. \end{aligned}$$

We know that $\tau _k< n\le \tau _{k+1}$ a.s. $|X_{\tau _k}|=|X_{n_0}|+k$, $|X_{\tau _{k+1}}|=|X_{n_0}|+k+1$ a.s. and $|X_n|\ge |X_{n_0}|+k$ a.s. Moreover, between times $\tau _k$ and $\tau _{k+1}$, the distance can increase with no more than $\tau _{k+1}-\tau _k$, and we have

$$\begin{aligned} \frac{|X_{n_0}|+k}{\tau _{k+1}}\le \frac{|X_n|}{n}\le \frac{|X_{n_0}|+k+(\tau _{k+1}-\tau _k)}{\tau _k},\quad \text {almost surely}. \end{aligned}$$

Since $\frac{|X_{\tau _k}|}{\tau _k}\cdot \frac{\tau _k}{\tau _{k+1}}=\frac{k}{\tau _{k+1}}$, together with Eq. (24) and the facts that $\frac{\tau _{k}}{\tau _{k+1}}\rightarrow 1$ and $\frac{|X_{n_0}|}{\tau _{k+1}}\rightarrow 0$ almost surely, we obtain that the left-hand side of the equation above converges to *l* almost surely, as $k\rightarrow \infty$. For the right-hand side, we use again Eq. (24), together with the fact that $\frac{\tau _{k+1}-\tau _k}{\tau _k}\rightarrow 0$ almost surely, since the increments $(\tau _{k+1}-\tau _k)$ are i.i.d and almost surely finite. This yields the almost sure convergence of the right-hand side of the equation above to the constant *l*, which implies that $\frac{|X_n|}{n}\rightarrow l$ almost surely as $n\rightarrow \infty$. $\square$

## Rotor Walks on Galton–Watson Trees

The methods we have used in proving the law of large numbers and the existence of the rate of escape for rotor walks $(X_n)$ with random initial configuration on regular trees can be, with minor modifications, adapted to the case when the rotor walk $(X_n)$ moves initially on a Galton–Watson tree ${\mathcal {T}}$. We get very similar results to the ones on regular trees, which we will state below. We will not write down the proofs again, but only mention the differences which appear on Galton–Watson trees.

Let ${\mathcal {T}}$ be a Galton–Watson tree with offspring distribution $\xi$ given by $p_k={\mathbb {P}}[\xi =k]$, for $k\ge 0$, and we assume that $p_0=0$, that is ${\mathcal {T}}$ is supercritical and survives with probability 1. Moreover, the mean offspring number $\mu ={\mathbb {E}}[\xi ]$ is also greater than 1. We recall the notation and the main result from [6]. For each $k\ge 0$ we choose a probability distribution ${\mathcal {Q}}_k$ supported on $\{0,\ldots ,k\}$. That is, we have the sequence of distributions $({\mathcal {Q}}_k)_{k\in \mathbb {N}_0}$, where

$$\begin{aligned} {\mathcal {Q}}_k = \big (q_{k,j}\big )_{0\le j\le k} \end{aligned}$$

with $q_{k,j}\ge 0$ and $\sum _{j=0}^k q_{k,j} = 1$. Let ${\mathcal {Q}}$ be the infinite lower triangular matrix having ${\mathcal {Q}}_k$ as row vectors, i.e.,

$$\begin{aligned} {\mathcal {Q}} = \begin{pmatrix} q_{00} &{}\quad 0 &{}\quad 0 &{}\quad 0 &{}\quad \ldots \\ q_{10} &{}\quad q_{11} &{}\quad 0 &{}\quad 0 &{}\quad \ldots \\ q_{20} &{}\quad q_{21} &{}\quad q_{22} &{}\quad 0 &{}\quad \ldots \\ q_{30} &{}\quad q_{31} &{}\quad q_{32} &{}\quad q_{33} &{}\quad \ldots \\ \vdots &{}\quad \vdots &{}\quad \vdots &{}\quad \vdots &{}\quad \ddots \\ \end{pmatrix}. \end{aligned}$$

Below, we write $\mathrm {d}_x$ for the (random) degree of vertex *x* in ${\mathcal {T}}$.

### Definition 5.1

A random rotor configuration $\rho$ on ${\mathcal {T}}$ is ${\mathcal {Q}}$-distributed, if for each $x\in {\mathcal {T}}$, the rotor $\rho (x)$ is a random variable with the following properties:

1. $\rho (x)$ is ${\mathcal {Q}}_{{\mathsf {d}}_x}$ distributed, i.e., ${\mathbb {P}}[\rho (x) = \mathrm {d}_x - l \,|\, \mathrm {d}_x = k] = q_{k,l}$, with $l=0,\ldots \mathrm {d}_x$,
2. $\rho (x)$ and $\rho (y)$ are independent if $x\not =y$, with $x,y\in {\mathcal {T}}$.

We write ${\mathsf {R}}_{{\mathcal {T}}}$ for the corresponding probability measure.

Then ${\mathsf {RGW}}=\mathsf {R}_{{\mathcal {T}}}\times {\mathsf {GW}}$ represents the probability measure given by choosing a tree ${\mathcal {T}}$ according to the ${\mathsf {GW}}$ measure, and then independently choosing a rotor configuration $\rho$ on ${\mathcal {T}}$ according to $\mathsf {R}_{{\mathcal {T}}}$. Recall that to the root $r\in {\mathcal {T}}$, we have added an additional sink vertex *s*. If we start with *n* rotor particles, one after another, at the root *r* of ${\mathcal {T}}$, with random initial configuration $\rho$, and we denote by $E_n({\mathcal {T}},\rho )$ the number of particles out of *n* that escape to infinity, then the main result of [6] is the following.

### Theorem 5.2

[6, Theorem 3.2] Let $\rho$ be a random ${\mathcal {Q}}$-distributed rotor configuration on a Galton–Watson tree ${\mathcal {T}}$ with offspring distribution $\xi$, and let $\nu = \xi \cdot {\mathcal {Q}}$. Then we have for ${\mathsf {RGW}}$-almost all ${\mathcal {T}}$ and $\rho$:

- $\displaystyle E_n({\mathcal {T}},\rho ) = 0$ for all $n\ge 1$, if ${\mathbb {E}}[\nu ] \le 1$,
- $$\displaystyle \lim _{n\rightarrow \infty } \frac{E_n({\mathcal {T}},\rho )}{n} = \gamma ({\mathcal {T}})$$, if $${\mathbb {E}}[\nu ] > 1$$,

where $\gamma ({\mathcal {T}})$ represents the probability that simple random walk started at the root of ${\mathcal {T}}$ never returns to *s*.

Let us denote $m:={\mathbb {E}}[\nu ]$. That is, if $m\le 1$, then $(X_n)$ is recurrent and if $m>1$, then $(X_n)$ is transient.

### Range and Speed on Galton–Watson Trees

**Tree of good children.** If $\rho$ is ${\mathcal {Q}}$-distributed,

$$\begin{aligned} {\mathbb {P}}[x \text { has }l\text { good children} \,|\, \mathrm {d}_x = k] = q_{k,l}, \end{aligned}$$

then the distribution of the number of good children of a vertex *x* in ${\mathcal {T}}$ is given by

25 $$\begin{aligned} {\mathbb {P}}[x \text { has }l\text { good children}] = \sum _{k = l}^{\infty } p_k q_{k,l}=:r_l, \end{aligned}$$

which is the $l{\text {th}}$ component of the vector $\nu = \xi \cdot {\mathcal {Q}}$. The tree of good children ${\mathcal {T}}^{\mathsf {good}}$ is in this case a Galton–Watson tree with offspring distribution $\nu = \xi \cdot {\mathcal {Q}}$ whose mean was denoted by *m*. Denote by $f_{{\mathcal {T}}}$ the generating function of ${\mathcal {T}}^{\mathsf {good}}$ and by *q* its extinction probability. For the range $R^{{\mathcal {T}}}_n$ of rotor walks $(X_n)$ on Galton–Watson trees, we get the following result.

#### Theorem 5.3

Let ${\mathcal {T}}$ be a Galton–Watson tree with offspring distribution $\xi$ and mean offspring number ${\mathbb {E}}[\xi ]=\mu >1$. If $(X_n)$ is a rotor walk with random ${\mathcal {Q}}$-distributed initial configuration on ${\mathcal {T}}$, and $\nu =\xi \cdot {\mathcal {Q}}$, then there is a constant $\alpha _{{\mathcal {T}}}>0$ such that

$$\begin{aligned} \lim _{n\rightarrow \infty }\frac{|R^{{\mathcal {T}}}_n|}{n}=\alpha _{{\mathcal {T}}},\quad {\mathsf {RGW}}\text { -almost surely}. \end{aligned}$$

If we write $m={\mathbb {E}}[\nu ]$, then the constant $\alpha _{{\mathcal {T}}}$ is given by:

(i) If $(X_n)_{n\in \mathbb {N}}$ is positive recurrent, then
  $$\begin{aligned} \alpha _{{\mathcal {T}}}=\frac{\mu -1}{2(\mu -m)}. \end{aligned}$$
(ii) If $(X_n)_{n\in \mathbb {N}}$ is null recurrent, then
  $$\begin{aligned} \alpha _{{\mathcal {T}}}=\frac{1}{2}. \end{aligned}$$
(iii) If $(X_n)_{n\in \mathbb {N}}$ is transient, then conditioned on the non-extinction of ${\mathcal {T}}^{\mathsf {good}}$,
  $$\begin{aligned} \alpha _{{\mathcal {T}}}=\frac{q-f_{{\mathcal {T}}}'(q)(q^2-q+1)}{q^2+q-f_{{\mathcal {T}}}'(q)(2q^2-q+1)} \end{aligned}$$
  where $q>0$ is the extinction probability of ${\mathcal {T}}^{\mathsf {good}}_d$.

The proof follows the lines of the proof of Theorem 1.1, with minor changes which we state below. The offspring distribution of the tree of good children ${\mathcal {T}}^{\mathsf {good}}$ will be here replaced with (25), and the corresponding mean offspring number is *m*. If $L_k$ and ${\mathcal {R}}_{\tau _k}$ are the same as in the proof of Theorem  3.2, then in case when $(X_n)$ moves on a Galton–Watson tree ${\mathcal {T}}$,

26 $$\begin{aligned} L_k = 1 + \sum _{v\in {\mathcal {R}}_k} \xi _v - |{\mathcal {R}}_k|,\quad \text {almost surely}, \end{aligned}$$

where $\xi _v$ denotes the (random) number of children of the vertex $v\in {\mathcal {T}}$. Since ${\mathcal {T}}$ is the initial Galton–Watson tree with mean offspring number $\mu$, by Wald’s identity we get

$$\begin{aligned} {\mathbb {E}}[L_1] =1+ \frac{1}{1-m} \mu - \frac{1}{1-m} = \frac{\mu - m}{1-m}:=\lambda >1. \end{aligned}$$

Then $\nu$ in Theorem 3.2 will be replaced with $\lambda$ and the rest works through. In the proof of Theorem 1.1(i), $\alpha$ will be replaced with $\frac{\mu -1}{2(\mu -m)}$. Theorem 3.3 works as well here, with a minor change in Eq. (12), where the last term will be

$$\begin{aligned} \frac{1 + \sum _{v\in {\mathcal {R}}_{k-1}} \xi _v - |{\mathcal {R}}_{k}|}{\tau _{k-1}}, \end{aligned}$$

while the following relations stay the same. In the transient case, in the proof of Theorem 3.5, when computing the expectation ${\mathbb {E}}_{\mathsf {non}}[N_0]$, the degree $d-1$ has to be replaced by the offspring distribution $\xi$, which in terms of the generating function $f_{{\mathcal {T}}}$ of ${\mathcal {T}}^{\mathsf {good}}$, and its extinction probability *q* produces the same result.

If $(X_n)$ is an uniform rotor walk on the Galton–Watson tree ${\mathcal {T}}$, we have a particularly simple limit. In [6], it was shown that $(X_n)$ is recurrent if and only if $\mu \le 2$. Moreover, the tree of good children ${\mathcal {T}}^{\mathsf {good}}$ is a subcritical Galton–Watson tree with offspring distribution given by $\nu = \left( \sum _{k\ge l} \frac{1}{k+1}p_k\right) _{l\ge 0}$, and mean offspring number $m = {\mathbb {E}}[\nu ] = \frac{\mu }{2} \le 1$.

#### Corollary 5.4

For the range of uniform rotor walks on Galton–Watson trees ${\mathcal {T}}$, we have

$$\begin{aligned} \lim _{n\rightarrow \infty }\frac{|R_n^{{\mathcal {T}}}|}{n} = \frac{\mu - 1}{\mu },\quad {\mathsf {RGW}}- \text { almost surely}. \end{aligned}$$

Finally, we also have the existence of the rate of escape.

#### Theorem 5.5

Let ${\mathcal {T}}$ be a Galton–Watson tree with offspring distribution $\xi$ and mean offspring number ${\mathbb {E}}[\xi ]=\mu >1$. If $(X_n)$ is a rotor walk with random ${\mathcal {Q}}$-distributed initial configuration on ${\mathcal {T}}$, and $\nu =\xi \cdot {\mathcal {Q}}$, then there exists a constant $l_{{\mathcal {T}}}\ge 0$, such that

$$\begin{aligned} \lim _{n\rightarrow \infty }\frac{|X_n|}{n} =l_{{\mathcal {T}}},\quad {\mathsf {RGW}}-\text {almost surely}. \end{aligned}$$

(i) If $(X_n)_{n\in \mathbb {N}}$ is recurrent, then $l_{{\mathcal {T}}}=0$.
(ii) If $(X_n)_{n\in \mathbb {N}}$ is transient, then conditioned on non-extinction of ${\mathcal {T}}^{\mathsf {good}}$,
  $$\begin{aligned} l_{{\mathcal {T}}}=\frac{(q-f'(q))(1-q)}{q+q^2-f'(q)(2q^2-q+1)}, \end{aligned}$$
  where $q>0$ is the extinction probability of ${\mathcal {T}}^{\mathsf {good}}_d$.

The constant $l_{{\mathcal {T}}}$ is in the following relation with the constant $\alpha _{{\mathcal {T}}}$ from Theorem 5.3 in the null recurrent and in the transient case:

$$\begin{aligned} 2\alpha _{{\mathcal {T}}}-l_{{\mathcal {T}}}=1. \end{aligned}$$

### Simulations on Galton–Watson Trees

We present here some simulation data about the growth of the range for a few one parameter families of Galton–Watson trees, for which the rotor walk is either recurrent or transient, depending on the parameter. In the table below, the Galton–Watson trees we use in the simulation are presented.

**Fig. 2:**
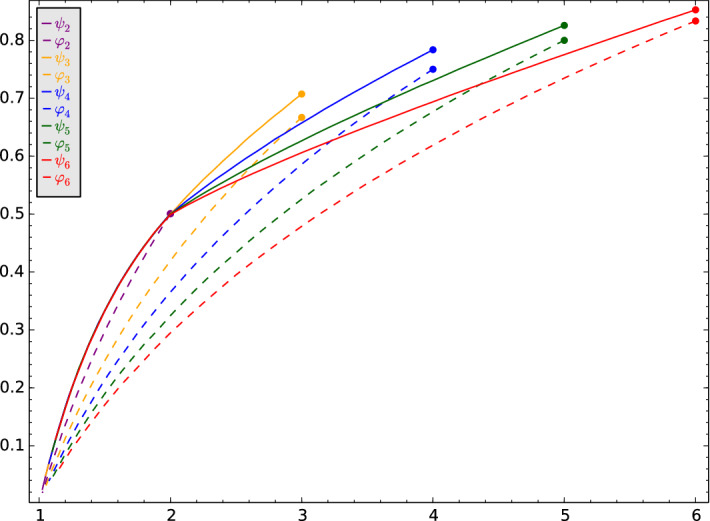
Plots of the linear growth coefficients of the size of the range for rotor walk (solid lines) and simple random walk (dashed lines) on the Galton–Watson trees ${\mathcal {T}}_2,\ldots ,{\mathcal {T}}_6$. The *x*-axis depicts the branching number of the tree. The dots show the corresponding values for the regular trees

**Table:** 

${\mathcal {T}}_i$	$(p_1,\ldots , p_6)$	$\mu _i$
${\mathcal {T}}_{2}$	$p_1 = p, p_2 = 1-p$	$2-p$
${\mathcal {T}}_{3}$	$p_1 = p, p_3 = 1-p$	$3-2 p$
${\mathcal {T}}_{4}$	$p_1 = p, p_4 = 1-p$	$4-3 p$
${\mathcal {T}}_{5}$	$p_1 = p, p_5 = 1-p$	$5-4 p$
${\mathcal {T}}_{6}$	$p_1 = p, p_6 = 1-p$	$6-5 p$

For each $i=2,\ldots ,6$ and $p\in [0,1)$, denote by ${\widetilde{\psi }}_i(p)$ and ${\widetilde{\varphi }}_i(p)$ the simulated values of the limits

27 $$\begin{aligned} \lim _{n\rightarrow \infty } \frac{| R^{i}_n|}{n} = {\widetilde{\psi }}_i(p) \qquad \lim _{n\rightarrow \infty } \frac{| S^{i}_n|}{n} = {\widetilde{\varphi }}_i(p), \end{aligned}$$

where $R_n^{i}$ and $S_n^{i}$ represent the range of the rotor walk and of the simple random walk up to time *n* on ${\mathcal {T}}_i$, respectively. To be able to compare the values, we plot the constants ${\widetilde{\psi }}_i(p)$ and ${\widetilde{\varphi }}_i(p)$ against the mean offspring number $\mu _i(p)$ of the offspring distribution on ${\mathcal {T}}_i$. That is, we look at the functions $\psi _i = {\tilde{\psi }}_i\circ \mu ^{-1}_i$ and $\varphi _i = {\tilde{\varphi }}_i\circ \mu ^{-1}_i$; see Fig. 2.

## Figures and Tables

**Fig. 3:**
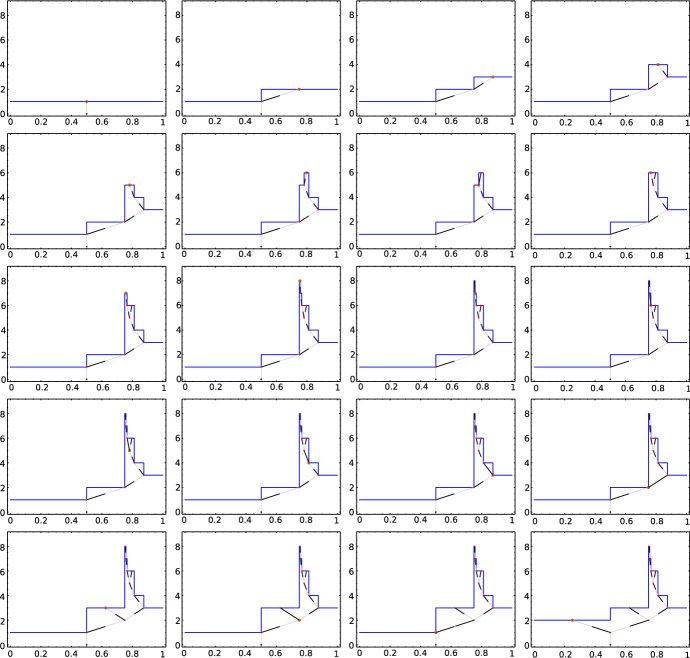
The first few steps in the computation of the range of rotor walk on ${\mathbb {T}}_2$. The blue functions are the contour functions of the current range $f_{R_n}$. The short black lines represent the current rotor configuration (Color figure online)

**Fig. 4:**
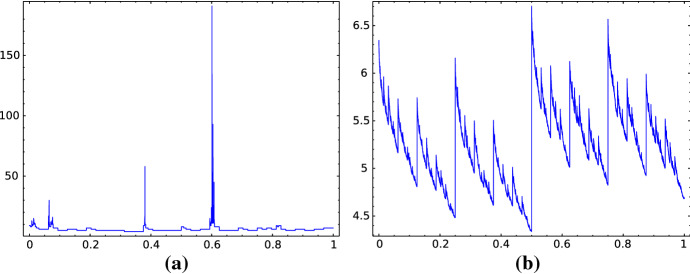
**a** Typical range contour of the uniform rotor walk on the binary tree ${\mathbb {T}}_2$. The rotor walk performed 10,000 steps. **b** Average over 1000 samples of the contour $f_{10{,}000}$ on the binary tree

**Fig. 5:**
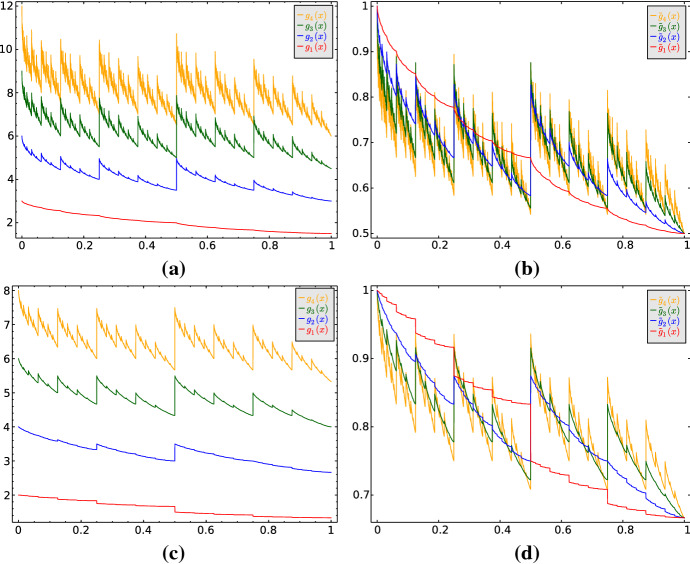
**a** Expected contours of the range of uniform rotor walk on ${\mathbb {T}}_2$ up to the completion of the first 4 excursions. **b** Normalized versions of the functions in **a**. **c** Expected contours $g_k$ for a positive recurrent rotor walk on ${\mathbb {T}}_2$ with $r_0 = 1/4$, $r_1 = 1/4$, $r_2 = 1/2$. **d** Scaled versions of the functions in **c**

## Appendix A: The Contour of a Subtree

We discuss here some further ways one can look at the range of rotor walks on regular trees ${\mathbb {T}}_d$, and at their contour functions, which according to simulations seem to have interesting fractal properties.

For $d\ge 2$, let $\Sigma _d = \{0,\ldots ,d-1\}$ and denote by $\Sigma _d^\star$ the set of finite words over the alphabet $\Sigma _d$. We use $\epsilon$ to denote the empty word. For $w\in \Sigma _d^\star$, we write |*w*| for the number of letters in *w*. If $w,v\in \Sigma _d^\star$, we write *wv* for the concatenation of the words *w* and *v*. We identify the tree ${\widetilde{{\mathbb {T}}}}_d$ with the set $\Sigma _d^\star$, since every vertex in ${\widetilde{{\mathbb {T}}}}_d$ can be uniquely represented by a word in $\Sigma _d^\star$. For $w = w_1\dots w_{n-1}w_n \in \Sigma _d^\star {\setminus }\{\epsilon \}$, the word $w_1\dots w_{n-1}$ is the predecessor of *w* in the tree, and for all $w\in \Sigma _d^\star$ the children of *w* are given by the words *wk* with $k \in \Sigma _d$. Using the previous notation, for $w=w_1\dots w_{n-1}w_n$ we have $w^{(0)} = w_1\dots w_{n-1}$ and for $k = 0,\ldots ,d-1$, $w^{(d-k)} = wk$. Let $A\subset {\widetilde{{\mathbb {T}}}}_d$ be a finite connected subset (a subtree) of ${\widetilde{{\mathbb {T}}}}_d$ containing the root $\epsilon$. We identify *A* with a piecewise constant function $f_A:[0,1]\rightarrow {{\mathbb {N}}}_{\ge 0}$ as following. For each $x\in [0,1]$, we identify *x* with the infinite word $x_1x_2\dots$ where the $x_i$ are the digits expansion of *x* in base *d*, that is $x = \sum _{i=1}^\infty x_i d^{-i}$. We then define the function $f_A$ pointwise as following

28 $$\begin{aligned} f_A(x) = \min \{n \ge 1: x_1\dots x_n \not \in A\}, \end{aligned}$$

and we call $f_A$ the *contour* of the set *A*.

### A.1: Contour of the Range: Recurrent Case

The range $R_n$ of the rotor walk $(X_n)$ on ${\mathbb {T}}_{d}$ is a subtree of ${\mathbb {T}}_{d}$. In what follows, we write $f_n = f_{R_n}$ for the contour of the range of the rotor walk up to time *n*. Since we start with a random initial rotor configuration, $f_n$ is a random càdlàg-function. See Fig. 3 for the contours of the rotor walk range for the first few steps of the process on the binary tree ${\mathbb {T}}_2$. Figure 4a shows a typical contour of the rotor range on the binary tree for $n = 10{,}000$ steps. Figure 4b shows a numerical approximation of the expectation ${\mathbb {E}}[f_{10000}]$.

As in the proofs of the law of large numbers for $|R_n|$, we look first at the times when the rotor walk returns to the root. Recall the definition of the return times $(\tau _k)$, as defined in (5), for recurrent rotor walks. Write again ${\mathcal {R}}_k = R_{\tau _k}$ for the range up to time $\tau _k$ of the *k*-th return of the rotor walk $(X_n)$ to the sink, and denote by $g_k(x) = {\mathbb {E}}\big [f_{{\mathcal {R}}_k}(x)\big ]$ the expected contour after the *k*-th excursion, that is $g_k(x) = \sum _{m=1}^\infty m {\mathbb {P}}\big [f_{{\mathcal {R}}_k}(x) = m\big ]$. Recall that we are in a case of a random initial configuration $\rho$ on ${\mathbb {T}}_d$, with ${\mathbb {E}}[\rho (v)]\ge 1$. The distribution of $\rho$ is ${\mathbb {P}}\big [\rho (v) = i\big ] = r_i\ge 0$. Some additional notation will be needed. For $i = 0,\ldots ,d$ let

$$\begin{aligned} p_i = \sum _{j=0}^{i-1} r_j, \qquad q_i = \sum _{j = i}^d r_j. \end{aligned}$$

For each $x = (x_1,x_2,\ldots ) \in [0, 1]$, we can now compute the probability ${\mathbb {P}}\big [f_{{\mathcal {R}}_1}(x) = m+1\big ]$ that the rotor walk visits the first *m* vertices of the ray represented by $(x_1,x_2,\ldots )$ before taking a step back toward the sink vertex. Once the rotor walk makes a step toward the sink, it cannot further explore the ray *x* without first returning to the sink vertex. Furthermore, the depth the walk can explore the ray before returning depends only on the initial rotor state along the vertices of the ray. Below, $f_{{\mathcal {R}}_1}(x) = 1$ means that $x_1$ is not in the range of the walk after the first full excursion, $x_1$ being a vertex at level 1. We get the following

$$\begin{aligned} {\mathbb {P}}\big [f_{{\mathcal {R}}_1}(x) = 1\big ]&= \left. {\left\{ \begin{array}{ll} r_1+r_2+\dots +r_d&{}\quad \text {if } x_1 = d-1, \\ r_2+\dots +r_d&{}\quad \text {if } x_1 = d-2, \\ &{}\quad \vdots \\ r_d&{}\quad \text {if } x_1 = 0 \end{array}\right. }\right\} = q_{(d-x_1)}.\\ {\mathbb {P}}\big [f_{{\mathcal {R}}_1}(x) = 2\big ]&= \left. {\left\{ \begin{array}{ll} r_0 &{} \quad \text {if } x_1 = d-1, \\ r_0+r_1 &{} \quad \text {if } x_1 = d-2, \\ &{} \quad \vdots \\ r_0+r_1+\dots +r_{d-1} &{} \quad \text {if } x_1 = 0 \end{array}\right. }\right\} \\&\;\;\cdot \left. {\left\{ \begin{array}{ll} r_1+r_2+\dots +r_d&{}\quad \text {if } x_2 = d-1, \\ r_2+\dots +r_d&{}\quad \text {if } x_2 = d-2, \\ &{}\quad \vdots \\ r_d&{}\quad \text {if } x_2 = 0 \end{array}\right. }\right\} \\&= p_{(d-x_1)} q_{(d-x_2)} \end{aligned}$$

For the general case $m\ge 1$, we get

29 $$\begin{aligned} {\mathbb {P}}\big [f_{{\mathcal {R}}_1}(x) = m\big ] = \left( \prod _{i = 1}^{m-1} p_{(d-x_i)}\right) \cdot q_{(d-x_m)}. \end{aligned}$$

After completing the $(m-1)$-st excursion, all rotors in the visited set ${\mathcal {R}}_{m-1}$ point toward the sink *o*. Hence, before completing the *m*-th excursion, the walk will visit all boundary points of ${\mathcal {R}}_{m-1}$. At each boundary point $w\in \partial {\mathcal {R}}_{m-1}$, the rotor walk makes a full excursion into the subtree rooted at *w* before exploring the rest of the boundary and finally returning to *o* to complete the excursion. Thus, the *m*-th excursion depends only on the set ${\mathcal {R}}_{m-1}$ and on the random initial states of the rotors on ${\mathbb {T}}_d{\setminus }{\mathcal {R}}_{m-1}$.

For $x = (x_1,x_2,\dots ) \in [0,1]$ denote by $\overleftarrow{x}^l = \{d^l x\} = (x_{l+1},x_{l+2},\dots )$, where $\{\bullet \}$ is the fractional part of a positive real number. For $k\ge 2$ we have ${\mathbb {P}}\big [f_{{\mathcal {R}}_k}(x) = 1\big ]=0$ since between each return the tree is explored at least for one additional level. When $k\ge 2$ and $m\ge 2$:

30 $$\begin{aligned} {\mathbb {P}}\big [f_{{\mathcal {R}}_k}(x) = m\big ] = \sum _{l=1}^{m-1} {\mathbb {P}}\big [f_{{\mathcal {R}}_1}(x) = l \big ]\cdot {\mathbb {P}}\big [f_{{\mathcal {R}}_{k-1}}(\overleftarrow{x}^l) = m-l\big ]. \end{aligned}$$

For $k\ge 2$ we have

$$\begin{aligned} g_k(x)&= {\mathbb {E}}\big [f_{{\mathcal {R}}_k}(x)\big ] = \sum _{m=1}^\infty m {\mathbb {P}}\big [f_{{\mathcal {R}}_k}(x) = m\big ]\\&= \sum _{m = 1}^\infty m \sum _{l=1}^{m-1} {\mathbb {P}}\big [f_{{\mathcal {R}}_1}(\overleftarrow{x}^l) = m-l\big ] \cdot {\mathbb {P}}\big [f_{{\mathcal {R}}_{k-1}}(x) = l\big ] \\&= g_{k-1}(x) + \sum _{l=1}^\infty g_1(\overleftarrow{x}^l) {\mathbb {P}}\big [f_{{\mathcal {R}}_{k-1}}(x) = l\big ]. \end{aligned}$$

Figure 5a shows the plots of the functions $g_1, \ldots , g_4$ for the uniform rotor walk on ${\mathbb {T}}_2$, which clearly suggests the fractal nature of these functions. Note that for $x\in \{0,1\}$ the shifted ray $\overleftarrow{x}^l$ always equals *x*. Hence, the number of steps the rotor walk descends into the left- and rightmost rays in the *k*-th excursion is i.i.d. In view of the two relations $g_k(0) = k g_1(0)$ and $g_k(1) = k g_1(1)$, it makes sense to look at the normalized versions ${\tilde{g}}_k(x) = \frac{g_k}{k}(x)$ (see Fig. 5b). Figure 5c, d shows corresponding plots for a positive recurrent rotor walk on ${\mathbb {T}}_2$ with initial distribution given by $r_0 = 1/4$, $r_1 = 1/4$ and $r_2 = 1/2$.

#### Theorem A.1

Set $g_0\equiv 0$. Then for all $x\in [0,1]$ and all $k\ge 1$, we have the self similar equations:

$$\begin{aligned} g_k\left( \frac{x+i}{d}\right) = 1 + \big (1-p_{(d-i)}\big ) g_{k-1}(x) + p_{(d-i)}g_k(x), \end{aligned}$$

for $$i\in \{0,\ldots ,d-1\}$$.

#### Proof

For $x\in [0,1]$ we identify *x* with its *d*-ary expansion $x=(x_1,x_2,x_3,\ldots )$, where $x = \sum _{i=1}^\infty x_i\cdot d^{-i}$ with $x_i \in \{0,\ldots ,d-1\}$. For $i\in \{0,\ldots ,d-1\}$ and $x=(x_1,x_2,x_3,\ldots )$, we have the correspondence

$$\begin{aligned} \frac{x+i}{d} = (i,x_1,x_2,x_3,\ldots ) \end{aligned}$$

and thus $\overleftarrow{\left( \frac{x+i}{d}\right) }^l = \overleftarrow{x}^{l-1}$ for all $l\ge 1$. It follows that ${\mathbb {P}}\big [f_{{\mathcal {R}}_1}\left( \frac{x+i}{d}\right) = 1\big ] = q_{(d-i)}$ and for all $m\ge 2$, as a consequence of (29)

$$\begin{aligned} {\mathbb {P}}\left[ f_{{\mathcal {R}}_1}\left( \frac{x+i}{d}\right) = m\right]&= p_{(d-i)}\left( \prod _{i=2}^{m-1}p_{(d-x_i)}\right) q_{(d-x_m)} \\&= p_{(d-i)} {\mathbb {P}}\big [f_{{\mathcal {R}}_1}(x)=m-1\big ]. \end{aligned}$$

We first look at the case $k=1$

$$\begin{aligned} g_1\left( \frac{x+i}{d}\right)&= {\mathbb {P}}\left[ f_{{\mathcal {R}}_1}\left( \frac{x+i}{d}\right) = 1\right] + \sum _{m\ge 2} m {\mathbb {P}}\left[ f_{{\mathcal {R}}_1}\left( \frac{x+i}{d}\right) = m\right] \\&=q_{(d-i)} + \sum _{m\ge 2} m p_{(d-i)}{\mathbb {P}}\big [f_{{\mathcal {R}}_1}(x)=m-1\big ] \end{aligned}$$

which in turn equals

$$\begin{aligned}&=q_{(d-i)} +p_{(d-i)}\sum _{m\ge 1} (m+1){\mathbb {P}}\big [f_{{\mathcal {R}}_1}(x)=m\big ] \\&=q_{(d-i)} +p_{(d-i)}\sum _{m\ge 1} m{\mathbb {P}}\big [f_{{\mathcal {R}}_1}(x)=m\big ] + p_{(d-i)}\sum _{m\ge 1} {\mathbb {P}}\big [f_{{\mathcal {R}}_1}(x)=m\big ] \\&= q_{(d-i)} +p_{(d-i)} + p_{(d-i)}g_1(x)=1 + p_{(d-i)}g_1(x). \end{aligned}$$

Since $g_0(x) = 0$, by definition the case $k=1$ follows. We now look at the case $k\ge 2$. By the convolution formula (30), we have

$$\begin{aligned}&{\mathbb {P}}\left[ f_{{\mathcal {R}}_k}\left( \frac{x+i}{d}\right) = m\right] \\&\quad = \sum _{l=1}^{m-1} {\mathbb {P}}\left[ f_{{\mathcal {R}}_1}\left( \frac{x+i}{d}\right) = l \right] \cdot {\mathbb {P}}\left[ f_{{\mathcal {R}}_{k-1}}\left( \overleftarrow{\left( \frac{x+i}{d}\right) }^l\right) = m-l\right] \\&\quad ={\mathbb {P}}\left[ f_{{\mathcal {R}}_1}\left( \frac{x+i}{d}\right) = 1 \right] \cdot {\mathbb {P}}\left[ f_{{\mathcal {R}}_{k-1}}\left( \overleftarrow{\left( \frac{x+i}{d}\right) }^1\right) = m-1\right] \\&\quad \quad +\sum _{l=2}^{m-1} {\mathbb {P}}\left[ f_{{\mathcal {R}}_1}\left( \frac{x+i}{d}\right) = l \right] \cdot {\mathbb {P}}\left[ f_{{\mathcal {R}}_{k-1}}\big (\overleftarrow{x}^{l-1}\big ) = m-l\right] \end{aligned}$$

which equals

$$\begin{aligned}&=q_{(d-i)}\cdot {\mathbb {P}}\left[ f_{{\mathcal {R}}_{k-1}}(x) = m-1\right] \\&\quad +p_{(d-i)}\sum _{l=2}^{m-1} {\mathbb {P}}\left[ f_{{\mathcal {R}}_1}(x) = l-1 \right] \cdot {\mathbb {P}}\left[ f_{{\mathcal {R}}_{k-1}}\big (\overleftarrow{x}^{l-1}\big ) = m-l\right] \\&=q_{(d-i)}\cdot {\mathbb {P}}\left[ f_{{\mathcal {R}}_{k-1}}(x) = m-1\right] \\&\quad +p_{(d-i)}\sum _{l=1}^{m-2} {\mathbb {P}}\left[ f_{{\mathcal {R}}_1}(x) = l \right] \cdot {\mathbb {P}}\left[ f_{{\mathcal {R}}_{k-1}}\big (\overleftarrow{x}^l\big ) = m-1-l\right] \\&=q_{(d-i)}\cdot {\mathbb {P}}\left[ f_{{\mathcal {R}}_{k-1}}(x) = m-1\right] +p_{(d-i)} {\mathbb {P}}\left[ f_{{\mathcal {R}}_k}(x) = m-1\right] , \end{aligned}$$

where in the last step we use the convolution formula (30) again in reverse. Thus,

$$\begin{aligned} g_k\left( \frac{x+i}{d}\right)&= \sum _{m=2}^\infty m {\mathbb {P}}\left[ f_{{\mathcal {R}}_k}\left( \frac{x+i}{d}\right) = m\right] =q_{(d-i)} \sum _{m=2}^\infty m {\mathbb {P}}\left[ f_{{\mathcal {R}}_{k-1}}(x) = m-1\right] \\&\quad + p_{(d-i)}\sum _{m=2}^\infty m {\mathbb {P}}\left[ f_{{\mathcal {R}}_k}(x) = m-1\right] \\&=q_{(d-i)} \sum _{m=1}^\infty (m+1) {\mathbb {P}}\left[ f_{{\mathcal {R}}_{k-1}}(x) = m\right] \\&\quad + p_{(d-i)}\sum _{m=1}^\infty (m+1) {\mathbb {P}}\left[ f_{{\mathcal {R}}_k}(x) = m\right] \\&= q_{(d-i)} \big (g_{k-1}(x) + 1\big ) + p_{(d-i)}\big (g_k(x) + 1\big ) \\&= 1 + (1-p_{(d-i)}) g_{k-1}(x) + p_{(d-i)}g_k(x), \end{aligned}$$

which proves the theorem in the general case. $\square$

**Some comments.** It may be interesting to understand on which graphs, other than regular and Galton–Watson trees, does the Einstein relation (1) hold. Other classes of trees such as periodic trees are definitely a good candidate.

One can also look at finer estimates such as law of iterated logarithm, or central limit theorems for the range and the speed for rotor walks on regular trees.
